# Light controlled self‐escape capability of non‐cationic carbon nitride‐based nanosheets in lysosomes for  hepatocellular carcinoma targeting stimulus‐responsive gene delivery

**DOI:** 10.1002/btm2.10558

**Published:** 2023-06-06

**Authors:** Ming‐Xuan Liu, Li Xu, Jia‐Yi Jiang, Hai‐Chen Dong, Peng‐Fei Zhu, Lei Cao, Jing Chen, Xiao‐Ling Zhang

**Affiliations:** ^1^ School of Pharmacy Nantong University Nantong China; ^2^ Institute of Translational Medicine, Medical College Yangzhou University Yangzhou Jiangsu P. R. China; ^3^ Jiangsu Key Laboratory of Integrated Traditional Chinese and Western Medicine for Prevention and Treatment of Senile Diseases Yangzhou University Yangzhou Jiangsu P. R. China

**Keywords:** CN‐based nanosheets, gene delivery, gene therapy, light controlled, non‐cationic carrier

## Abstract

High positive charge‐induced toxicity, easy lysosomal degradation of nucleic acid drugs, and poor lesion sites targeting are major problems faced in the development of gene carriers. Herein, we proposed the concept of self‐escape non‐cationic gene carriers for targeted delivery and treatment of photocontrolled hepatocellular carcinoma (HCC) with sufficient lysosome escape and multiple response capacities. Functional DNA was bound to the surface of biotin‐PEG_2000_‐modified graphitic carbon nitride (Bio‐PEG‐CN) nanosheets to form non‐cationic nanocomplexes Bio‐PEG‐CN/DNA. These nanocomposites could actively target HCC tissue. Once these nanocomplexes were taken up by tumor cells, the accumulated reactive oxygen species (ROS) generated by Bio‐PEG‐CN under LED irradiation would disrupt the lysosome structure, thereby facilitating nanocomposites escape. Due to the acidic microenvironment and lipase in the HCC tissue, the reversible release of DNA could be promoted to complete the transfection process. Meanwhile, the fluorescence signal of Bio‐PEG‐CN could be monitored in real time by fluorescence imaging technology to investigate the transfection process and mechanism. In vitro and in vivo results further demonstrated that these nanocomplexes could remarkably upregulate the expression of tumor suppressor protein P53, increased tumor sensitivity to ROS generated by nanocarriers, and realized effective gene therapy for HCC via loading *P53* gene.

## 1 INTRODUCTION

Gene therapy realizes the purpose of cancer treatment via introducing therapeutic nucleic acids to modulate the expression of target proteins.^1^, ^2^, ^3^, ^4^ However, the negatively charged surface of nucleic acid drug prevents its passive diffusion across the anionic cell membrane because of electrostatic repulsion.^5^, ^6^, ^7^ Therefore, the construction of high‐efficiency gene vectors is the key to cancer gene therapy. Considering nucleic acid surface electrical properties, cationic carriers have been extensively exploited because they can efficiently load the nucleic acids drugs and deliver them into target cells via electrostatic interaction.^8^, ^9^, ^10^, ^11^ Furthermore, gene delivery efficiency is closely related to the cationic charge density of the carrier.^12^ Higher positive charge densities can produce a stronger proton sponge effect, which is more favorable for carrier escape from lysosomes to improve the transfection efficiency.^13^ However, the toxicity caused by the positive charge density greatly hinders the clinical application of cationic carriers in gene therapy.^14^, ^15^ Recently, the emergence of non‐cationic carriers has addressed the problem of charge toxicity.^16^, ^17^ It should be noted that the poor lysosome escape ability of non‐positively charged carriers leads to the destruction of nucleic acid drugs by the abundant enzymes there, reducing the efficiency of gene therapy. Therefore, enhancing the lysosomal escape capacity of non‐cationic carriers is critical for cancer gene therapy.

Some explorations have been carried out on improving the lysosome escape of non‐cationic carriers, and part of carriers exhibited certain tumor gene therapy effects.^18^ However, not enough work has been reported on non‐cationic carriers, and most of which are passively targeted to tumor sites to the best of our knowledge. Along this line, we proposed an active HCC‐targeted gene therapy strategy to selectively identify and kill hepatoma cells via designing non‐cationic gene carriers with lysosomal escape function. Researches have indicated that the overexpression of biotin receptors in tumors is much higher than that in normal tissues, and the molecular structure of biotin is relatively simple and easy to functionalize.^19^, ^20^ Therefore, biotin (vitamin B7 or vitamin H) was used as an active HCC‐targeting molecule in this study.

Carbon nitride (CN or named g‐C_3_N_4_) has the advantages of easy modification, unique optical properties, and outstanding biocompatibility, endowing it with extensive attention in the biomedical field.^21^, ^22^, ^23^, ^24^ In particular, CN‐based materials (CNs) are capable of generating reactive oxygen species (ROS) through photolysis of water.^25^, ^26^, ^27^, ^28^ When CNs were captured by lysosomes, the ROS generated by CNs could disrupt the membrane structure of lysosome, thereby facilitating their escape from lysosomes.^29^, ^30^ In addition, nanomaterials have the natural advantage of passive accumulation in the liver, which can increase the enrichment of drugs in the liver.^31^ Our previous preliminary studies further showed that CN nanosheets have a certain degree of nucleic acid delivery function for the first time.^17^ Therefore, CN nanosheets were chosen as the core of gene delivery. At present, there were the following challenges in the application of CNs in nucleic acid delivery: (1) the poor dispersibility of CNs in water and easy to aggregate; (2) the insufficient agglomeration effect of CNs on DNA.

**SCHEME 1:**
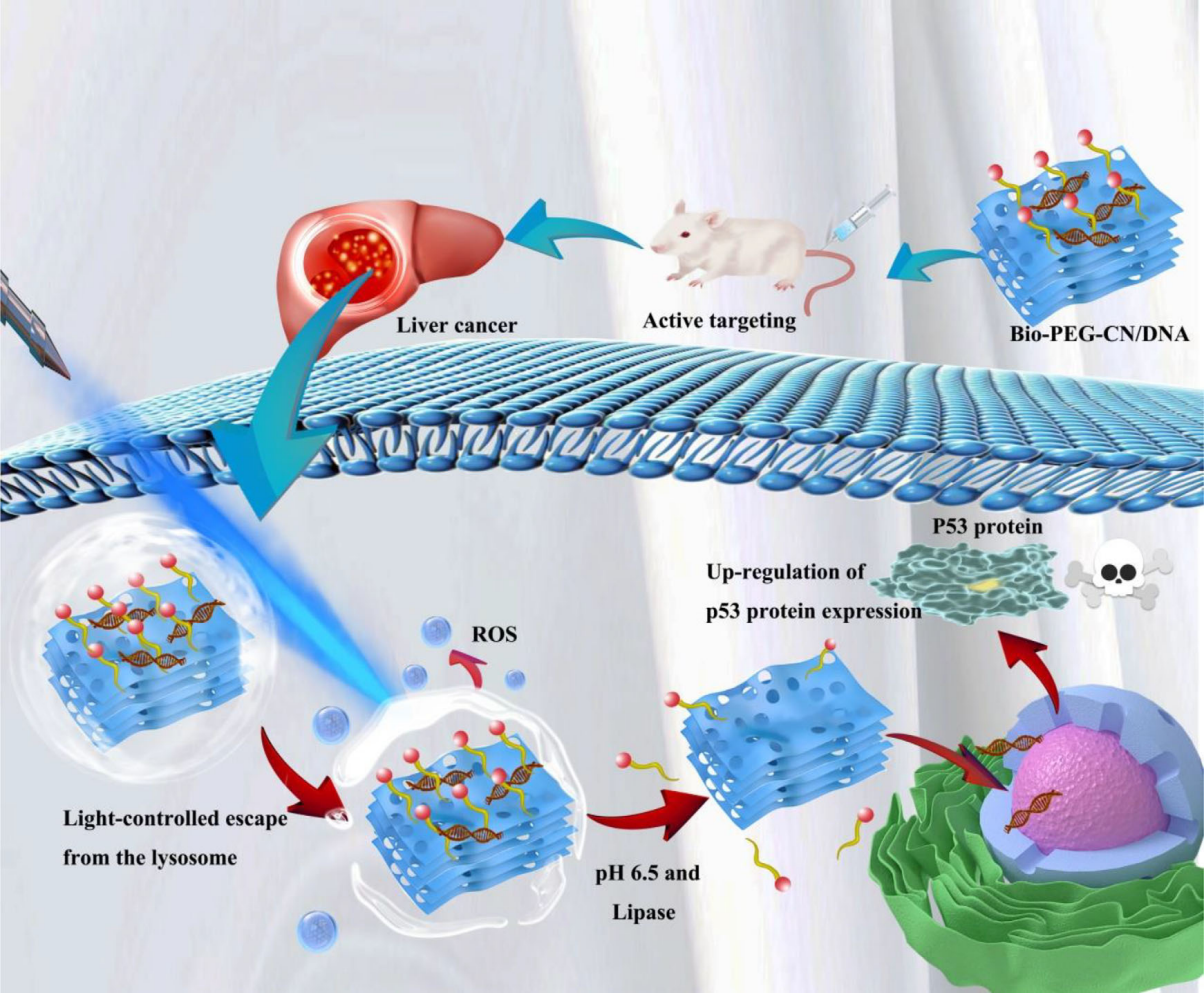
Schematic illustration of Bio‐PEG‐CN/DNA nanocomplexes in HCC therapy. The ROS generated by the LED irradiation of Bio‐PEG‐DNA nanocomposites would destroy the structure of lysosome and promote the escape of nanocomposites from lysosomes. The tumor microenvironment would promote the release of *P53* gene and up‐regulated the expression of P53 protein, thereby realizing HCC gene therapy.

In this contribution, we designed novel non‐cationic nanosheets (Bio‐PEG‐CN) by integrating biotin with CNs via stimuli‐responsive lipid bonds. Bio‐PEG‐CN nanosheets were able to escape from lysosome after LED irradiation to realize efficient gene delivery and HCC therapy (Scheme 1). Traditional non‐cationic carriers face the disadvantages of being trapped by lysosomes, poor tumor targeting, and difficulty in nucleic acid drug release. Different from traditional non‐cationic carriers, the Bio‐PEG‐CN could generate an appropriate amount of ROS under light irradiation to facilitate its escape from lysosomes. The introduction of biotin‐PEG_2000_ (Bio‐PEG_2000_) enabled the nanosheets to actively target the HCC site and enhanced the dispersibility of the nanosheets in water. The Bio‐PEG_2000_ group combined with the fluorescence imaging capability of CN nanosheets was also conducive to the specific identification of tumor sites via fluorescence imaging technology. In addition, the linking unit lipid bonds could promote the stimulatory response of Bio‐PEG‐CN nanosheets to the acidic microenvironment (pH ≈ 4.0–6.0) and lipase at HCC site,^32^, ^33^ and realized the reversible release of nucleic acid drugs. The released *P53* gene could induce the expression of the corresponding protein to achieve HCC gene therapy.^34^, ^35^ In the present study, Bio‐PEG‐CN nanosheets were prepared and utilized as light‐induced self‐escape from lysosomes, multiple‐responsive non‐positively charged gene carriers. The Bio‐PEG‐CN/*P53* nanocomplexes further activated the *P53*‐induced apoptosis signal by delivering the *P53* gene, and induced the ROS generation via LED irradiation, effectively realizing the gene therapy for HCC in nude mice.

## 2 MATERIALS AND METHODS

### 2.1 Materials

*N, N′* dicyclohexylcarbodiimide (DCC) and 4‐Dimethylaminopyridine (DMAP) were purchased from Tokyo Chemical Industry (TCI, Japan). Melamine was purchased from Energy Chemical (Shanghai, China). Bio‐PEG_2000_ was purchased from Aladdin Biochemical Technology Co., Ltd (Shanghai, China). Gold view II, pUC‐18 DNA, tris–HCl, Lyso‐Tracker Green, MTT, and loading buffer were purchased from Solarbio Science & Technology Co., Ltd. (Beijing, China). Sarcosine oxidase, bromocresol green, uricase, and vanadate were purchased from Sigma‐aldrich (USA). EGFP‐DNA was purchased from Clontech (USA). *P53*‐DNA and DNA‐Cy5 were purchased from Ruibiotech Co., Ltd. (Beijing, China). PVDF membranes were purchased from Millipore (USA).

### 2.2 Preparation of the bulk CN

The bulk CN was prepared by polymerization of melamine molecules at high temperature. Five grams of melamine was placed in a crucible and calcined at 600°C for 2 h under nitrogen atmosphere. The heating rate was 5°C/min, and the temperature was maintained at 600°C for 2 h. After the combustion products were cooled to room temperature, the bulk CN powder was obtained by performing grinding.

### 2.3 Preparation of CN–COOH nanosheets

First, 1 g bulk CN was added to the mixture of concentrated nitric acid (20 mL) and sulfuric acid (20 mL), and the reaction was stirred for 2 h at room temperature. The mixture was then diluted with deionized 500 mL water and washed for several times, and the obtained white product was the CN–COOH nanosheets.

### 2.4 Preparation of Bio‐PEG‐CN nanosheets

Bio‐PEG‐CN nanosheets were prepared by esterification reaction using flake CN–COOH as the original material. First, 10 mg CN–COOH and 30 mg Bio‐PEG were added to 5 mL DMSO and sonicated for 30 min to obtain a clear solution. Then, 10 mg DCC and 2.5 mg DMAP were added to the mixed solution. The reaction was stirred at room temperature for 48 h. After the end of the reaction, dialysis was performed through a dialysis bag for 12 h to remove small molecules. The obtained Bio‐PEG‐CN was diluted to 1 mg/mL in ultrapure water and sonicated for 8 h. The final Bio‐PEG‐CN nanosheets were stored at 4°C.

### 2.5 Gel retardation assay

Different concentrations of CN or Bio‐PEG‐CN nanosheets were mixed with 4 μL Trips–HCl (50 mM, pH 7.4) and 0.9 μL *pUC‐18* (200 μg mL^−1^) in the ultrapure water. The total volume of the mixture was 20 μL. After assembling the mixture for 40 min at 37°C, 2 μL loading buffer (10×) was added. The mixture was then electrophoresed on a 0.7% (w/v) agarose gel containing GelRed in TAE running buffer at 85 V for 40 min. Finally, the electrophoresis results were visualized by the Vilber Lourmat system.

### 2.6 Intracellular ROS determination

HepG2 cells were seeded in Glass Bottom Cell Culture Dishes at a concentration of 8000 cells per well and cultured in complete medium for 24 h. Then, 40 μg mL^−1^ Bio‐PEG‐CN nanosheets were incubated with cells for 4 h. After changing the medium, the treated cells were irradiated via the LED for 0–15 min and incubated for 12 h. The medium was changed and cells were treated with 10 μL of DCFH‐DA probe for 30 min. Finally, the treated cells were washed 3–5 times with PBS, and the fluorescence signal was detected via confocal laser scanning microscope (CLSM).

### 2.7 Cytotoxicity assay

Cells were seeded into a 96‐well plate at a concentration of 5000 cells/well, then 150 μL of complete medium was added and cultured for 24 h. Cells were then incubated with different concentrations of Bio‐PEG‐CN nanosheets for 48 h; 20 μL MTT (5 mg/mL) was added to each well, and after standing for 4 h, the medium was replaced with 200 μL DMSO. Optical density (OD) was recorded at 490 nm using a Thermo Scientific Multiskan GO. The viability of the cells was calculated as cell viability = [(OD_samples_ − OD_DMSO_)/(OD_control_ − OD_DMSO_)] × 100%.

### 2.8 Cell imaging

HepG2 cells were seeded in Glass Bottom Cell Culture Dishes at 2000 cells per dish and cultured for 24 h; 40 μg mL^−1^ Bio‐PEG‐CN and 5 μg DNA‐Cy5 were added to the EP tube containing 600 μL DMEM and mixed at 37°C for 30 min. The prepared DNA complex solution was added to the cells to replace the medium, and incubated in an incubator at 37°C, 5% CO_2_ for the corresponding time. Finally, these cells were washed 3–5 times with PBS, and observed via CLSM.

### 2.9 In vitro DNA transfection

HepG2, L‐02, and H22 cells were seeded in Glass Bottom Cell Culture Dishes at a concentration of 8 × 10^4^ cells/well, respectively and cultured in complete medium for 24 h in a 37°C incubator; 40 μg mL^−1^ Bio‐PEG‐CN and 10 μg mL^−1^ EGFP plasmid DNA were added to the EP tube containing 600 μL DMEM and mixed at 37°C for 30 min. The prepared DNA complex solution was added to the cells to replace the medium, and incubated in an incubator at 37°C, 5% CO_2_ for 4 h. Then, the DNA complex solution was replaced with 1 mL complete medium and incubated for 24 h. Finally, these treated cells were observed via CLSM.

### 2.10 Intracellular colocalization studies

HepG2 cells were seeded in Glass Bottom Cell Culture Dishes at 2000 cells per dish and cultured for 24 h. The cells were then incubated with Bio‐PEG‐CN/DNA nanocomposites and LysoTracker in a 37°C incubator for 30 min, followed by LED irradiation for 10 min. The Bio‐PEG‐CN/DNA‐treated group without LED irradiation was used as a control. Finally, the medium was replaced with PBS and the treated cells were observed via CLSM.

### 2.11 Cell‐uptake mechanistic studies

HepG2 cells were seeded in Glass Bottom Cell Culture Dishes at a concentration of 8 × 10^4^ cells/well and cultured in complete medium for 24 h in a 37°C incubator. HepG2 cells were pre‐incubated with 2.5 mM MβCD, 75 μM AM, 50 μM CPZ, or 50 μM GE for 1 h, and then 1 mL DMEM containing DNA‐Cy5 complexes was added to the cells. After 2 h of incubation, Cy5‐positive cells were tested by flow cytometric analysis to explore the pathways for uptake of DNA complexes.

### 2.12 Animals and tumor models

All animal studies were approved by the Animal Ethical and Welfare Committee (AEWC) prior to experimentation. The approved protocol number for animal studies was SCXK (Su 2022‐0009). All animal studies complied with ARRIVE guidelines. Female BALB/c athymic nude mice were obtained from the Animal Center of Nantong University and raised under standard conditions. The animals were housed in sterile cages within laminar airflow hoods in a specific pathogen‐free room and fed with autoclaved food and water. It was reported that differences in various physiological indicators of hormone circulation levels in female mice were close to male mice in many animal models.^36^ Even individual differences in certain sex‐differentiated phenotypes were more pronounced in male mice, such as androgen levels affecting tumor treatment.^37^ In addition, female nude mice were widely used in tumors treatment experiments.^38^, ^39^ The xenograft mouse model was established via subcutaneously inoculating 5 × 10^6^ cancer cells. Tumor volume in nude mice was determined by measuring the length (L) and the width (W) and using the formula *V* = 1/2 × *LW* ^2^. The tumor volume grew to approximately 80–90 mm^3^ before in vivo experiment.

### 2.13 In vivo biodistribution study

HepG2 bearing nude female mice (*n* = 3) were injected intravenously with Bio‐PEG‐CN/DNA‐Cy5 ([Bio‐PEG‐CN] = 2.5 mg/kg, [DNA‐Cy5] = 5 μg). Fluorescence signal was monitored via an in vivo imaging system with appropriate wavelength (λ_ex_ = 650 nm, λ_em_ = 670 nm). To study the biodistribution of Bio‐PEG‐CN/DNA nanocomplexes, tumor‐bearing nude mice were randomly divided into three groups. Nude mice were then injected intravenously with Bio‐PEG‐CN/DNA nanocomplexes through the tail vein. Monitoring was performed at 6, 9, 12, and 24 h (*n* = 3) after administration. The mice were then euthanized and their hearts, livers, spleens, lungs, kidneys, and tumors were collected.

### 2.14 In vivo pharmacodynamic study

When the tumor volume was approximately 80–90 mm^3^, nude mice were randomly assigned to four groups (*n* = 6): (a) PBS control, (b) Bio‐PEG‐CN nanosheets ([Bio‐PEG‐CN] = 5 mg/kg) under light radiation, (c) Bio‐PEG‐CN/*P53* ([Bio‐PEG‐CN] = 5 mg/kg, [*P53*] = 10 μg) under dark condition, and (d) Bio‐PEG‐CN/*P53* ([Bio‐PEG‐CN] = 5 mg/kg, [*P53*] = 10 μg) under light radiation. Nude mice were injected every 3 days for a total of seven cycles. Tumor size and mouse weight were measured and calculated at each injection. Nude mice were sacrificed 2 days after the seventh treatment, and tumors were dissected according to institutional guidelines. Throughout the experiment, each mouse was designated and tracked individually. In addition, tumors were cut into small pieces, fixed with 40% formalin, and then embedded in the paraffin. Histopathological analysis of tissue sections was performed by H&E staining. The treatment effect was evaluated by comparing the experimental and the control groups.

### 2.15 Western blot analysis

Total protein was extracted from tumor tissues of nude mice. Equal amounts of solubilized proteins were separated by 10%–15% SDS‐PAGE and transferred onto PVDF membranes. After blocking, the strips were incubated with *P53* primary antibody overnight at 4°C. After incubation with corresponding secondary antibody, enhanced chemiluminescence plus reagents were added and the membrane was exposed. The emitted light was captured by a BioSpectrum 410 multispectral imaging system equipped with a Chemi 410 HR camera and analyzed via Gel‐Pro Analyzer Version 5.0.

### 2.16 In vivo biosafety study

Healthy female BALB/c mice were used to assess the biosafety of Bio‐PEG‐CN/*P53*. Mice were divided into two groups (*n* = 5). The mice were injected with Bio‐PEG‐CN/*P53* ([Bio‐PEG‐CN] = 5 mg/kg, [*P53*] = 10 μg). Unmedicated mice were used as control. The blood was collected for blood biochemistry analysis. Blood biochemistry indicators were analyzed by combining blood samples with sarcosine oxidase (Sigma‐Aldrich), bromocresol green (Sigma‐Aldrich), uricase (Sigma‐Aldrich), and vanadate (Sigma‐Aldrich), respectively. For liver function indexes, aspartate aminotransferase, alanine aminotransferase, γ‐glutamyl transferase, and alkaline phosphatase were measured via automatic biochemical analyzer including UV‐MDH method, UV‐LDH method, L‐γ‐glutamyl‐3‐carboxy‐4‐nitrmethod and sodium 4‐nitrophenyl phosphate method, respectively.

### 2.17 Statistical analysis

All experiments were repeated with at least three independent samples, and each measurement was performed in triplicates. If the normal distribution was evident, a one‐way ANOVA was performed. The data were displayed as average values.

## 3 RESULT AND DISCUSSION

### 3.1 Characterization of Bio‐PEG‐CN

**FIGURE 1:**
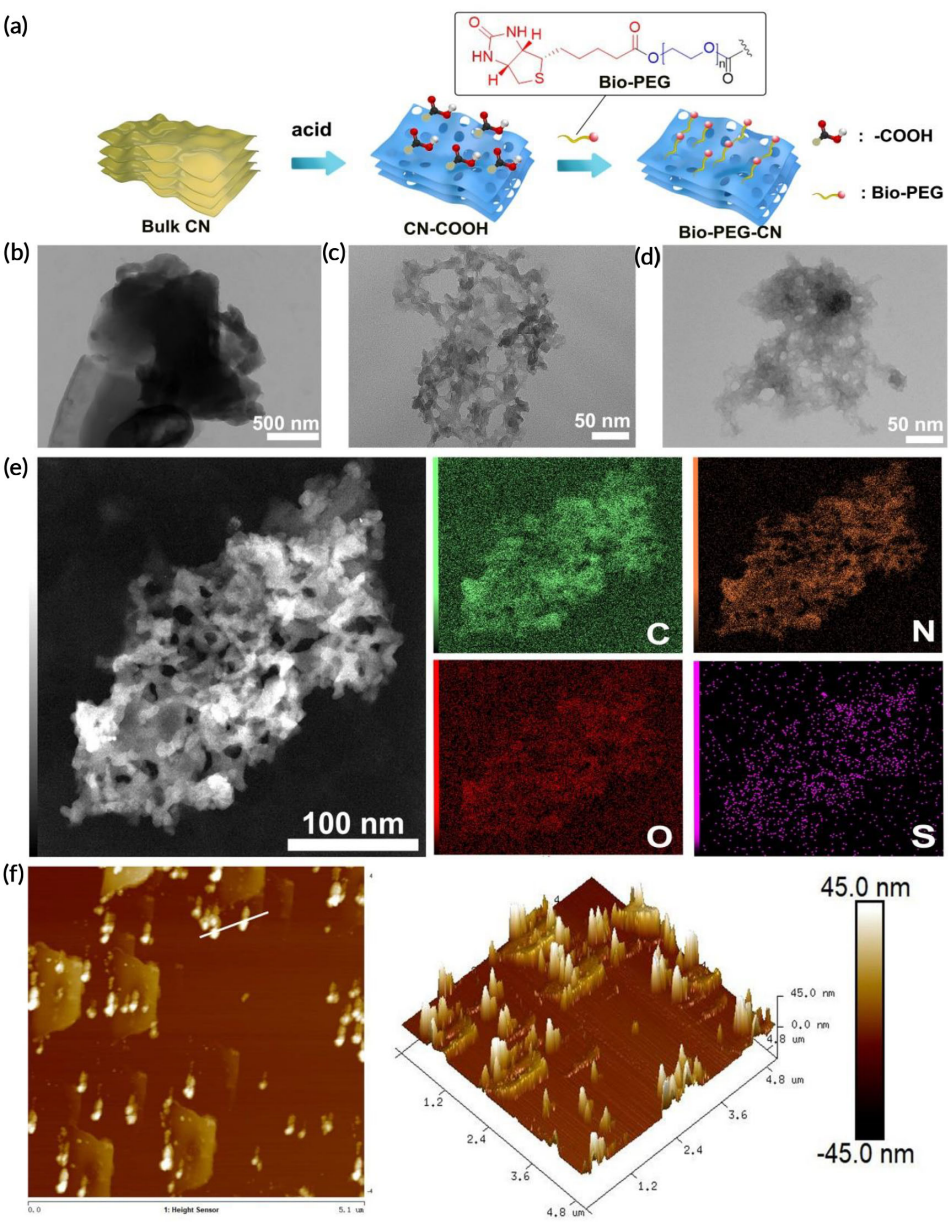
(a) Preparation method of Bio‐PEG‐CN. TEM image of (b) bulk CN, (c) CN–COOH, and (d) Bio‐PEG‐CN nanosheets. (e) Elemental mapping images of Bio‐PEG‐CN. (f) AFM images of Bio‐PEG‐CN nanosheets.

As shown in Figure 1a, Bio‐PEG‐CN nanosheets were mainly synthesized through solid‐phase synthesis, acidification, and esterification reactions. Bulk CN could be obtained via reacting melamine in solid phase at high temperature. Then, through further acidification reaction, –COOH unit was introduced to obtain porous CN–COOH nanosheets. New absorption bands at 2700 (O–H stretching vibration of –COOH) and 1770 cm^−1^ (C=O stretching vibration of –COOH) in the FTIR spectrum of CN–COOH nanosheets indicated successful modification of COOH units (Figure S1). Finally, the target nanosheets Bio‐PEG‐CN were prepared by introducing the targeting unit Bio‐PEG_2000_ and the stimuli‐responsive unit lipid bond through an esterification reaction. According to the XRD analysis, Bio‐PEG‐CN nanosheets existed only one stacked peak of conjugated aromatic structure at 27.4°, indicating that the atomic structure of CN was largely preserved after various modifications (Figure S2).

The morphologies and sizes of Bio‐PEG‐CN nanosheets were further characterized utilizing TEM, FTIR, AFM, DLS, etc. As shown in Figure 1b,c, unlike bulk CN, numerous clear pore‐like structures were found in the CN–COOH nanosheets after acid treatment via TEM measurements. The appearance of pore‐like structure could improve the adsorption performance of this material.^40^ Due to the modification of the material by Bio‐PEG_2000_, the pore‐like structure and edges of the final obtained Bio‐PEG‐CN nanosheets became blurred (Figure 1d). The FTIR spectra of Bio‐PEG‐CN nanosheets showed new absorption bands around 3450 (overtone peak of C=O in ester bond), 1650 (stretching vibration of C=O in ester bond), and 1050 cm^−1^ (C–O–C in ester bond), further indicating the successfully introduction of Bio‐PEG units through the esterification reaction (Figure S1). The OH base peak (2700, O–H stretching vibration of –COOH) in the COOH on the surface of the CN nanosheets was also found to have completely disappeared through FTIR spectra characterization of Bio‐PEG‐CN. Therefore, it could be concluded that COOH was completely modified by Bio‐PEG_2000_. The elemental composition and distribution of Bio‐PEG‐CN nanosheets were obtained via elemental mapping. As presented in Figure 1e, the expected elements including C (from CN nanosheets), N (from CN nanosheets), O (from the lipid bond and PEG_2000_), and the S (from biotin unit) were found in the Bio‐PEG‐CN nanosheets, which achieved uniform distribution in the CN‐based nanosheets. Furthermore, the UV–Vis absorption of Bio‐PEG‐CN exhibited a distinct blue shift compared to CN–COOH (Figure S3). The reason for this phenomenon might be caused by the introduction of Bio‐PEG_2000_ units. The above results indicated that Bio‐PEG units were successfully modified on CN nanosheets.

**FIGURE 2:**
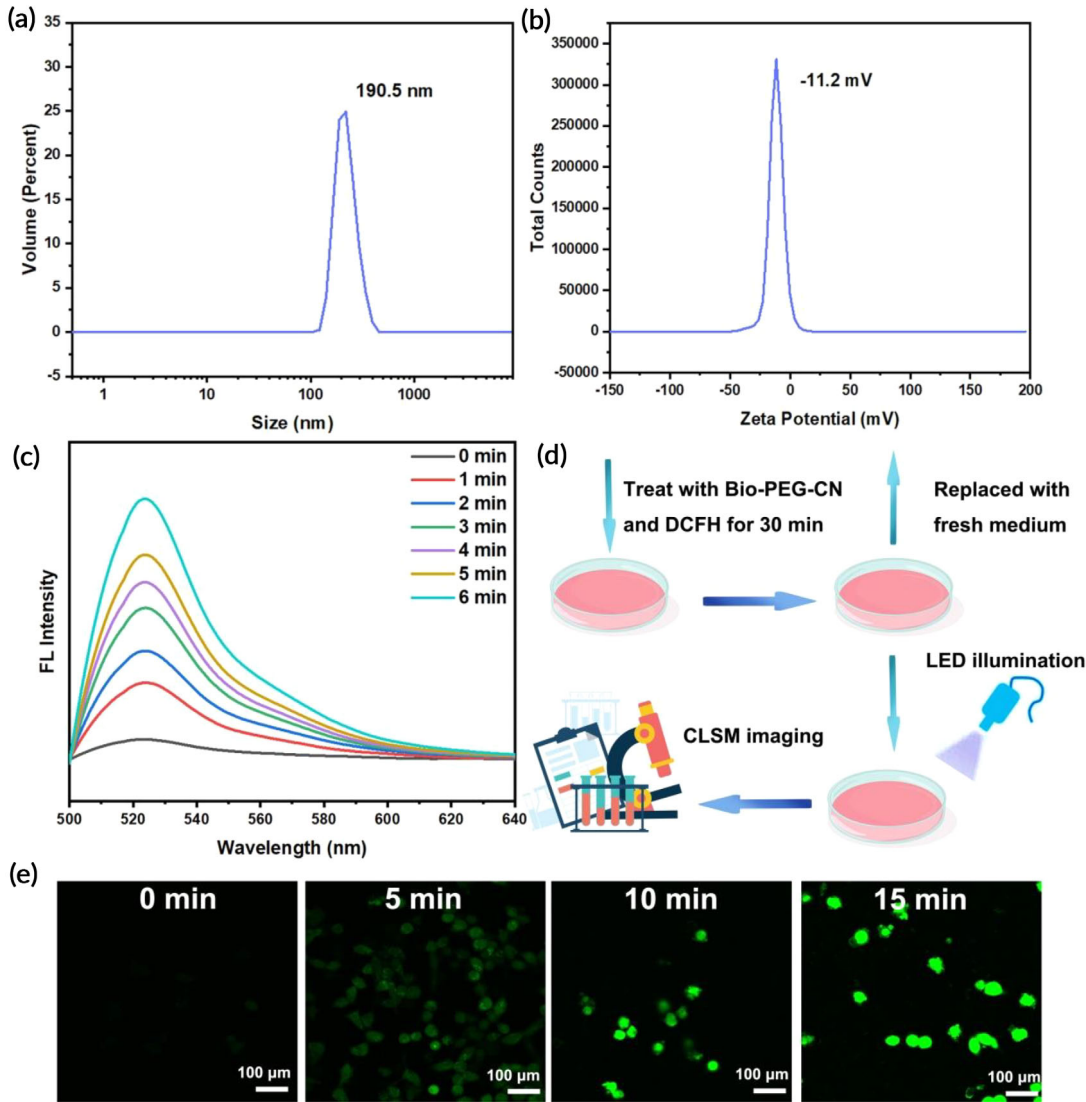
(a) DLS size distribution of Bio‐PEG‐CN nanosheets. (b) Zeta potentials of Bio‐PEG‐CN in Trips buffer (pH 7.4). (c) Fluorescence spectra of the mixture of DCFH and Bio‐PEG‐CN nanosheets (10 μg mL^−1^). (d) Schematic illustration of intracellular ROS generation monitoring. (e) Confocal laser scanning microscope (CLSM) images of HepG2 cells treated with Bio‐PEG‐CN nanosheets at different irradiation times. CLSM imaging was captured at an emission wavelength of 495–566 nm. Scale bars: 100 μm, LED wavelength: 401 nm, power density: 450 mW/cm^2^.

In addition, Bio‐PEG‐CN nanosheets were able to emit strong fluorescence emission (~425 nm) under UV light excitation (Figure S4 and S5), which was beneficial for fluorescence tracking. The mechanism of gene drug delivery could be studied more deeply, exploiting this property. Notably, the lateral thickness of Bio‐PEG‐CN nanosheets was about 40 nm (Figure 1f and Figure S6), and their average size was about 190.5 nm (Figure 2a). This appropriate size distribution of Bio‐PEG‐CN nanosheets could further facilitate cellular endocytosis.^41^, ^42^ The results of Zeta potential indicated that the surface of these nanosheets retained a certain degree of negative charge, which could avoid the cytotoxicity caused by the high positive charge (Figure 2b). Therefore, these nanosheets had potential as gene carriers with visualization functions.

### 3.2 ROS generation

Recently, ROS generation has been demonstrated to facilitate the escape of nanocarriers from lysosomes. Therefore, 2′,7′‐dichlorofluorescein (DCFH) was used to evaluate the light‐induced ROS capability of Bio‐PEG‐CN nanosheets, which would be oxidized by ROS to produce green fluorescence emission.^43^ As shown in Figure 2c, the mixed solution of DCFH and Bio‐PEG‐CN nanosheets showed time‐dependent fluorescence enhancement under LED illumination. Furthermore, the production of ROS in HepG2 cells after Bio‐PEG‐CN treatment was also investigated via the ROS index probe DCFH‐DA (Figure 2d,e and Figure S7). It could be seen that HepG2 cells treated with Bio‐PEG‐CN nanosheets exhibited brighter fluorescence signals with the prolongation of LED irradiation time, indicating a significant generation of ROS. Intracellular and extracellular results were consistent.

### 3.3 DNA condensation and release

**FIGURE 3:**
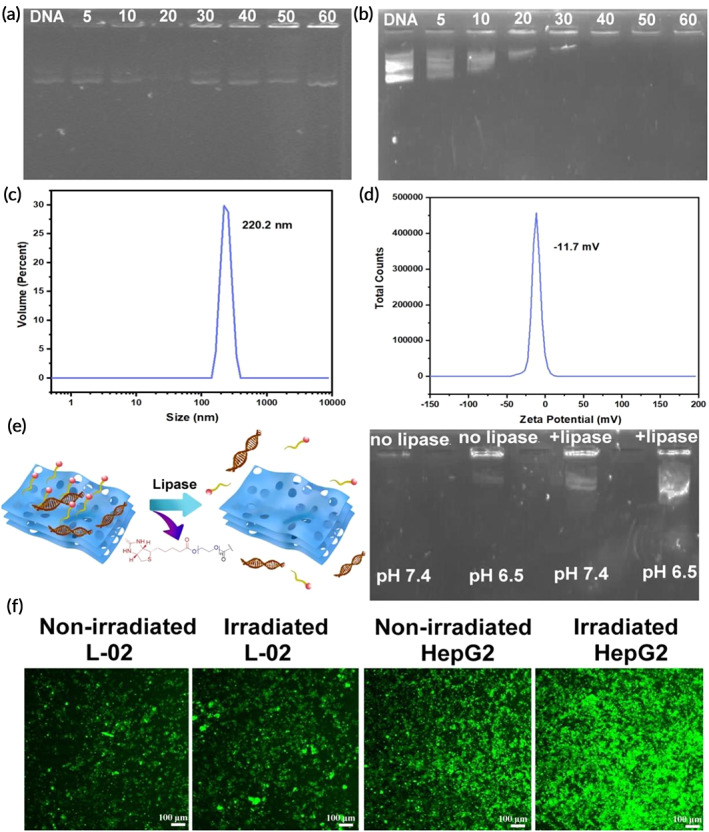
Aggregation of pUC‐18 DNA via (a) CN–COOH and (b) Bio‐PEG‐CN at different concentrations (μg mL^−1^). (c) DLS size distribution of Bio‐PEG‐CN/DNA and (d) Zeta potentials of Bio‐PEG‐CN/DNA in Trips buffer (pH 7.4). (e) Gel electrophoresis to measure the reversible release of DNA condensation induced by Bio‐PEG‐CN (40 μg mL^−1^) under different conditions. Incubation time: 1 h. (f) GFP expression CLSM imaging (10×) of Bio‐PEG‐CN/DNA in L‐02 and HepG2 cells. CLSM imaging was captured at an emission wavelength of 495–566 nm. (Bio‐PEG‐CN) = 40 μg mL^−1^, (EGFP plasmid DNA) = 10 μg mL^−1^, irradiation time: 10 min, incubation time: 24 h, scale bar: 100 μm.

To analyze the ability of Bio‐PEG‐CN nanosheets to condense DNA, the gel retardation assay was performed. As shown in Figure 3a, DNA was still not fully concentrated even at high concentrations of CN–COOH, which might be caused by the poor stability of CN–COOH in aqueous solution. However, the introduction of Bio‐PEG_2000_ group greatly enhanced the stability of the nanosheets in water, enabling 40 μg mL^−1^ Bio‐PEN‐CN to fully condense plasmid DNA via hydrogen bonding and π–π interactions (Figure 3b). Next, the encapsulation efficiency and loading capacity of plasmid DNA within the preformed Bio‐PEG‐CN nanosheets were investigated. After incubating Cy5‐labeled DNA with Bio‐PEG‐CN nanosheets at 37°C for 40 min, the supernatant was collected via centrifugation. The encapsulation efficiency (EE) was calculated by measuring the concentration change of DNA (EE% = (1 ─ concentration of DNA in the supernatant/concentration of initial DNA) × 100%).^44^ The encapsulation efficiency of Bio‐PEN‐CN/DNA was 72.4%, further indicating that Bio‐PEN‐CN could condense DNA to a certain extent. In addition, the Bio‐PEG‐CN nanosheets demonstrated observable encapsulation of DNA‐Cy5, in which a distinct blue color was observed in the complex particles (Figure S8). The blue color in the DNA‐Cy5 group without nanosheets was dispersed in solution. The sizes of Bio‐PEG‐CN/DNA were also tested utilizing DLS measurement. As shown in Figure 3c, the average particle sizes measured via DLS were 220.2 nm. It could be seen that the sizes of Bio‐PEG‐CN/DNA slightly increased after DNA condensation, due to DNA adsorption on the nanosheets. In addition, the Zeta potential of Bio‐PEG‐CN/DNA composite nanosheets was −11.7 mV, avoiding the positive charge toxicity (Figure 3d). The value of Zeta potential was also related to the stability of the material.^45^, ^46^, ^47^ The Zeta potential of Bio‐PEG‐CN/DNA nanocomplexes did not change much compared with Bio‐PEG‐CN, indicating that the stability of this material was not significant affected after loading DNA.

The introduction of ester bonds enabled Bio‐PEG‐CN nanosheets to be cleaved in acidic and lipase microenvironments to facilitate nucleic acid release. Then, the aggregation reversibility of Bio‐PEG‐CN/DNA complexes under mildly acidic and lipase conditions was investigated by gel electrophoresis experiments (Figure 3e). It could be seen that the complexes reversibly released part of the DNA at pH 6.5 or in the presence of lipase. Especially when these two conditions were met simultaneously, more DNA was released from the complexes. The liver, as a digestive organ, is rich in a large amount of lipase.^48^, ^49^, ^50^, ^51^ HepG2 cells also exhibit a relatively higher lipase activity and expression levels compared to the other colon cancer cell lines.^52^ According to gel electrophoresis experiments, better DNA release ability of the Bio‐PEG‐CN/DNA complex was found under the coexistence of lipase and weak acidity. In the presence of acidic tumor microenvironment and abundant lipase in hepatoma cells (HepG2), the lipid bonds of Bio‐PEG‐CN material could be hydrolyzed more rapidly to realize the release of nucleic acid drugs at the lesion site. The environment of the lesion site was simulated by lowering the pH and adding lipase in vitro experiments. The gene transfection effect of the nanosheets at liver cancer sites could be preliminarily inferred via evaluating the release of DNA from Bio‐PEG‐CN‐DNA composite nanosheets in the lesion simulation environment. Through the in vitro experiments, the Bio‐PEG‐CN/DNA complexes were found to have pH and enzyme‐stimulated response ability, which was beneficial to the reversible release of DNA at HCC sites.

### 3.4 DNA transfection in vitro

Prior to DNA delivery experiments, the cytotoxicity of Bio‐PEG‐CN nanosheets was assessed via MTT assays (Figure S9). After incubating different concentrations of Bio‐PEG‐CN nanosheets in hepatoma cells HepG2 for 24 h, the cell viability remained above 80% even at a high concentration of 60 μg mL^−1^. In addition, normal hepatocytes L‐02 also exhibited more than 80% viability after incubation with these nanosheets at a concentration of 40 μg mL^−1^. The higher survival rate of HepG2 cells might be due to the different growth activities of tumor cells and normal cells. Therefore, Bio‐PEG‐CN nanosheets showed good enough biocompatibility for the next step of DNA transfection.

The gene transfection efficiency of Bio‐PEG‐CN nanosheets was evaluated using green fluorescence protein gene (EGFP‐DNA). As shown in Figure 3f, the expression of GFP was detected by CLSM. EGFP‐DNA was transfected into HepG2 cells via Bio‐PEG‐CN nanosheets more efficiently than L‐02 cells due to the tumor cell targeting of Bio‐PEG units. The green fluorescence signal in HepG2 was significantly enhanced after LED irradiation via fluorescence intensity analysis, indicating that the ROS generated by Bio‐PEG‐CN nanosheets promoted the transfection of *EGFP*‐DNA (Figure S10). In addition, cell flow cytometry experiments were further used to quantitatively evaluate the gene transfection efficiency of Bio‐PEG‐CN nanosheets. The HepG2 LED irradiation group showed the highest transfection efficiency via cell flow cytometry testing with a transfection efficiency of up to 89.7% (Figure S11), which was consistent with the CLSM results. To further explore the gene transfection efficiency of Bio‐PEG‐CN in other types of cells, particularly in suspension cells, H22, a type of liver cancer cell isolated from mouse ascites, was also used for further transfection experiments. As shown in Figure S12, both CLSM and flow cytometry results indicated that these nanosheets exhibited efficient gene transfection efficiency after LED irradiation, which was able to reach 62.1%. It could be concluded that Bio‐PEG‐CN nanosheets had photoresponsive gene delivery in different types of cells, and the ROS generated by short‐time LED irradiation did not affect the function of DNA.

### 3.5 Light‐induced escape from the lysosome

**FIGURE 4:**
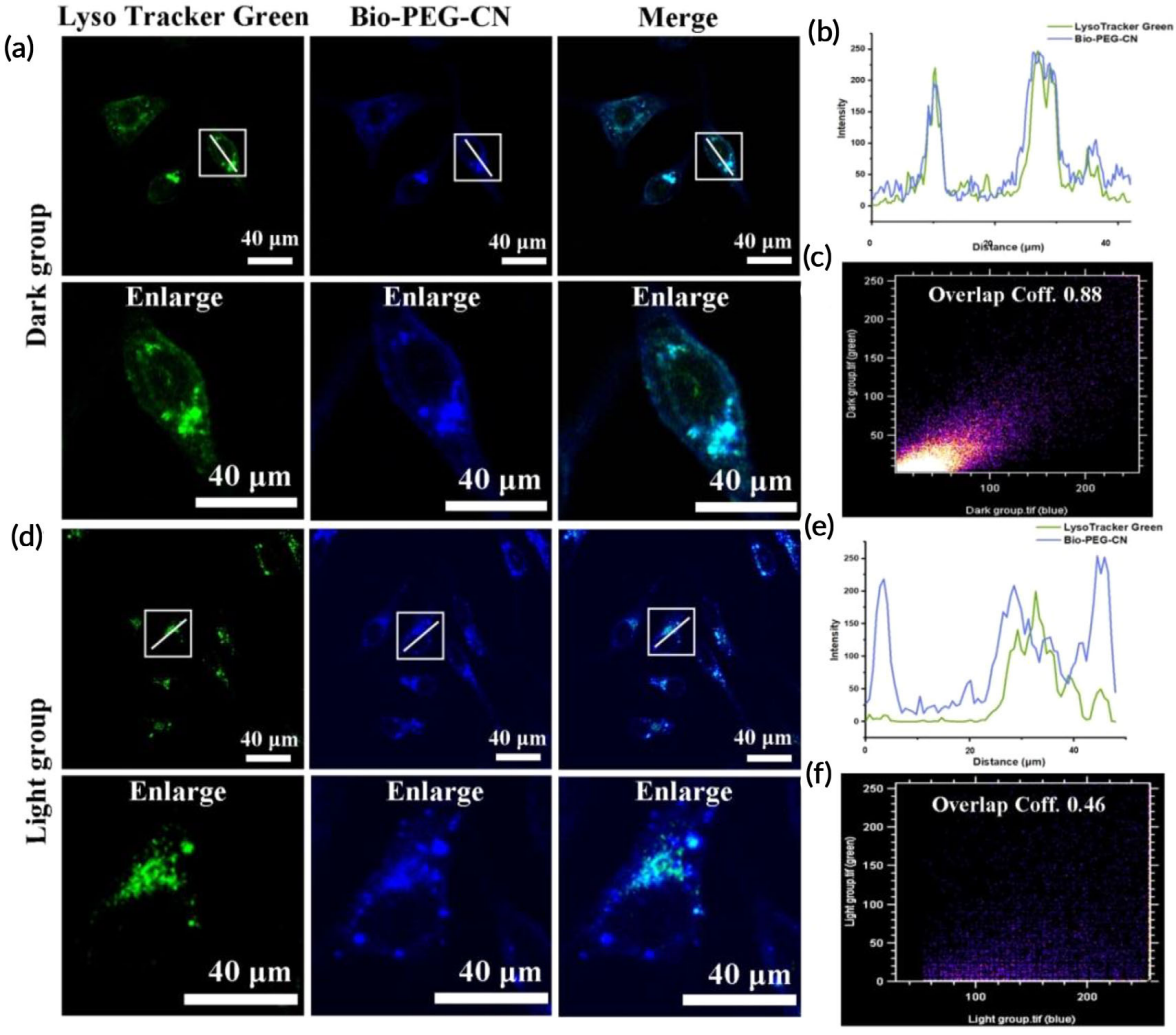
(a) CLSM images (40×) of colocalization with Bio‐PEG‐CN/DNA and Lyso‐Tracker Green in HepG2 under dark conditions. (b,c) Analysis of colocalization with Bio‐PEG‐CN/DNA and Lyso‐Tracker Green in HepG2 under dark conditions. (d) CLSM image of colocalization with Bio‐PEG‐CN/DNA and Lyso‐Tracker Green in HepG2 under LED illumination. CLSM imaging was captured at an emission wavelength of 495–566 nm and 400–450 nm. (e,f) Analysis of colocalization with Bio‐PEG‐CN/DNA and Lyso‐Tracker Green in HepG2 under LED illumination. Wavelength: 401 nm, power density: 450 mW/cm^2^, irradiation time: 10 min (Bio‐PEG‐CN) = 40 μg mL^−1^, scale bar: 40 μm.

The escape of non‐positively charged gene carriers from lysosomes is particularly important for successful gene delivery and therapy. As Bio‐PEG‐CN nanosheets exhibited the properties of fluorescence imaging, HepG2 cells were co‐cultured with Bio‐PEG‐CN/DNA and lysosomal tracker green to study the lysosome escape of this carrier. Lysosomes were observed via the green channel, and the Bio‐PEG‐CN/DNA was inspected via the blue channel. As shown in Figure 4a,b, co‐staining of Bio‐PEG‐CN and lysosome tracker revealed that the blue and green fluorescent signals overlapped well in dark conditions. The Pearson correlation coefficient of these two colors fluorescence signals was as high as 0.88 under dark, indicating the capture of Bio‐PEG‐CN/DNA by lysosomes (Figure 4c). It could be concluded that the absence of the proton sponge effect made it difficult for Bio‐PEG‐CN/DNA to escape rapidly from lysosomes. However, after irradiating Bio‐PEG‐CN/DNA‐treated cells with LED for 10 min, a substantial amount of Bio‐PEG‐CN fluorescence signal was separated from lysosomes (Figure 4d,e). This was further verified by a Pearson correlation coefficient of 0.46, indicating that ROS generated by LED irradiation promoted Bio‐PEG‐CN/DNA escape from lysosomes (Figure 4f).

### 3.6 Internalization of DNA complexes

**FIGURE 5:**
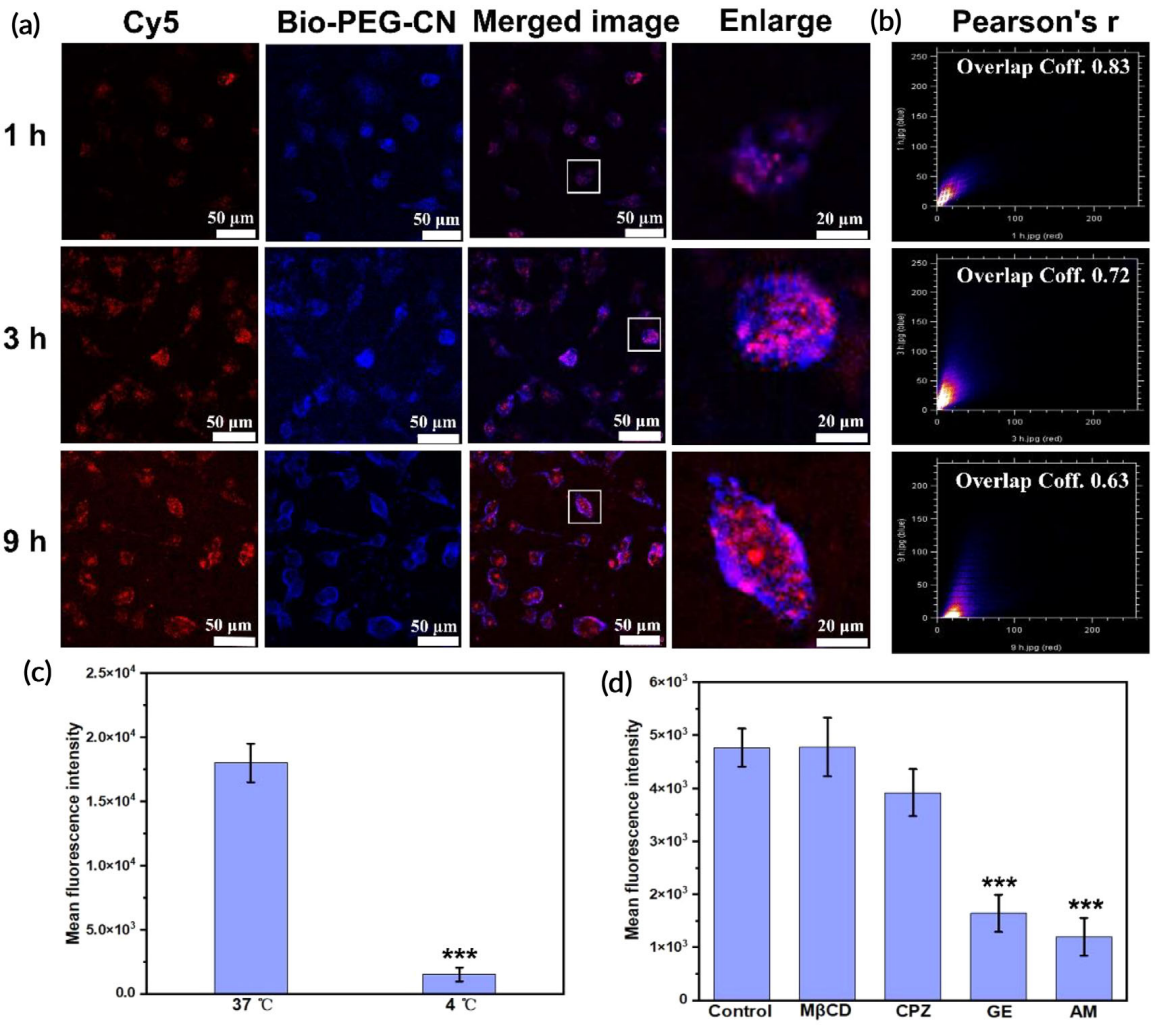
(a) CLSM images (40×) of HepG2 cells treated with Bio‐PEG‐CN/DNA for 1, 3, and 9 h, scale bar: 50 μm and 20 μm. CLSM imaging was captured at an emission wavelength of 660–700 nm and 400–450 nm. (b) Analysis of colocalization with DNA‐Cy5 and Bio‐PEG‐CN in HepG2 cells for 1, 3, and 9 h. (c,d) Inhibitory assays of Bio‐PEG‐CN/DNA cellular uptake under different conditions in HepG2 cells. HepG2 without any inhibitor served as a control. ***p < 0.001 versus the control group. [Cy5‐DNA] = 10 μg mL^−1^, [Bio‐PEG‐CN] = 40 μg mL^−1^.

The delivery process is critical to understanding nucleic acid transfection and developing a new generation of universal transfection reagents. The cellular uptake, gene release, and internalization patterns of the vector complexes require further investigation. Therefore, the internalization pathway of DNA complexes was further investigated via fluorescence imaging techniques. As shown in Figure 5a, part of DNA‐Cy5 (red channel) was delivered into cells by Bio‐PEG‐CN nanosheets (blue channel) in only 1 h. The intracellular fluorescence signals increased gradually in a time‐dependent manner, especially the red fluorescence of DNA‐Cy5, indicating that the Bio‐PEG‐CN/DNA complexes were easily taken up by HepG2 cells. In addition, the red and blue fluorescence signals in HepG2 gradually separated with increasing incubation time. The Pearson correlation coefficient of these two fluorescent signals was also reduced from 0.83 to 0.63 (Figure 5b). Therefore, it could be concluded that the DNA in the nanocarriers was reversibly released.

The internalization mode is a basic parameter for the DNA carrier, and that of Bio‐PEG‐CN/DNA complexes needs to be further explored. To further understand the endocytic mechanism of cellular uptake, Bio‐PEG‐CN/DNA complexes were incubated with HepG2 cells under different conditions. As shown in Figure 5c, the cellular uptake of Bio‐PEG‐CN/DNA complexes was significantly decreased at low temperature compared to 37°C, suggesting that the internalization of these complexes was at least partially realized by energy‐dependent endocytosis.^53^ It should be noted that endocytosis is considered to be the main route for non‐viral nanocarriers to enter cells, and it proceeds via different mechanisms: caveolae‐mediated endocytosis (CavME), clathrin‐mediated endocytosis (CME), and “lipid rafts”‐like endocytosis and micropinocytosis.^54^ Herein, genistein (GE, CavME inhibitor),^55^ amiloride hydrochloride (AM, macropinocytosis inhibitor),^53^ methyl‐β‐cyclodextrin (MβCD, “lipid rafts”‐like endocytosis inhibitor),^56^ and chlorpromazine (CPZ, CME inhibitor)^53^ were utilized to identify the main mechanism of cellular uptake for this system. As shown in Figure 5d, after the Bio‐PEG‐CN/DNA complexes were pre‐incubated with GE or AM at 37°C, the fluorescence signal in the cells decreased significantly. Based on these results, we could conclude that Bio‐PEG‐CN/DNA nanocomplexes were mainly taken up by cells through the CavME and macropinocytosis pathways. Due to the diversity of endocytosis, Bio‐PEG‐CN/DNA could enter into cells relatively quickly.

### 3.7 Active tumor targeting

**FIGURE 6:**
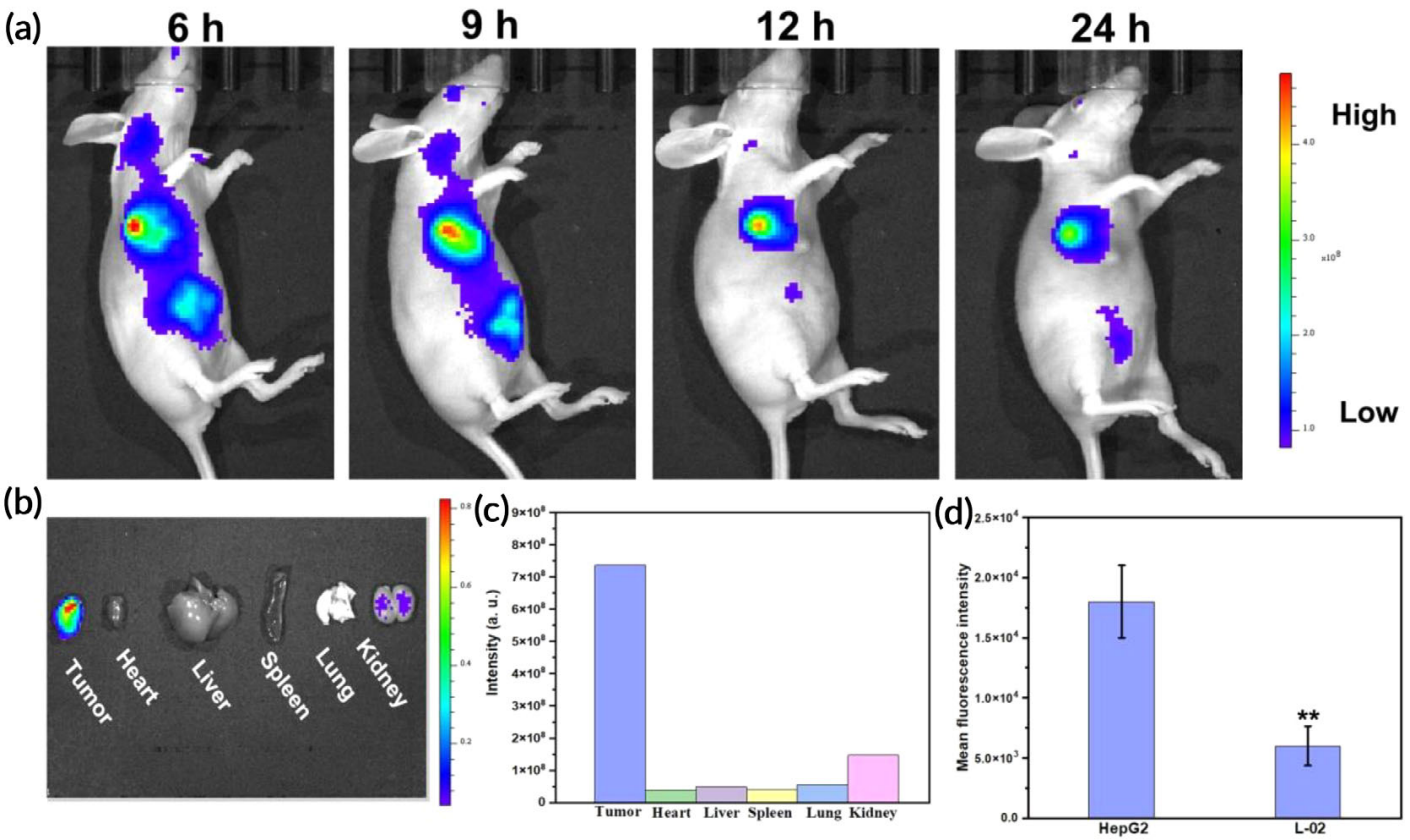
(a) In vivo imaging of nude mice after injection with Bio‐PEG‐CN/DNA ([Bio‐PEG‐CN] = 2.5 mg/kg, [DNA‐Cy5] = 5 μg) at different time points, respectively. (b) Ex vivo fluorescence imaging of resected tumors and organs at 24 h. (c) Fluorescence intensity analysis in tumors and organs of nude mice. (d) Cellular uptake of Bio‐PEG‐CN/DNA by HepG2 and L‐02 cells. **p < 0.01 versus the HepG2 group. [Bio‐PEG‐CN] = 40 μg mL^−1^, [EGFP plasmid DNA] = 10 μg mL^−1^.

According to the literature, the overexpression of biotin receptors in cancer cells was much higher than that in normal cells.^20^, ^57^, ^58^, ^59^, ^60^ As the hepatoma cell, large amounts of biotin receptors had also been reported in HepG2.^61^, ^62^, ^63^, ^64^ Therefore, biotin‐modified CN‐based material Bio‐PEG‐CN had the potential to deliver nucleic acids to liver tumors. To confirm that Bio‐PEG‐CN/DNA nanocomposites could realize HCC‐specific aggregation in vivo, HepG2‐cell xenografted nude mouse models was established. The nanocomplexes formed by the assembly of DNA‐Cy5 and Bio‐PEG‐CN nanosheets were injected intravenously into mice and then tracked via an animal imaging system. As shown in Figure 6a, the fluorescence signal of Bio‐PEG‐CN/DNA nanocomposites accumulated at the tumor and liver sites after 6 h, due to the introduction of the tumor targeting unit Bio‐PEG_2000_ and the natural enrichment of the nanomaterial to the liver.^31^ The fluorescence signal of these nanocomposites in the tumor still remained strong even at 12 h postinjection. Eventually, the fluorescence intensity in the tumor gradually decreased at 24 h after injection, which might be caused by the metabolism of nanocomplexes in vivo. Meanwhile, ex vivo fluorescence imaging of resected tumors and organs at 24 h were obtained, and the in vivo biodistribution of the nanocomplexes was assessed. As shown in Figure 6b,c, the Bio‐PEG‐CN/DNA nanocomposites were effectively enriched in the tumor, exhibiting strong fluorescence emission. In addition, the weak fluorescence signals present in nude mouse kidney further suggested that these nanocomplexes were able to be metabolized. Flow cytometry was also used to evaluate the targeting of Bio‐PEG‐CN/DNA nanocomplexes to hepatoma cells. It could be seen that the uptake of Bio‐PEG‐CN/DNA nanocomposites by the hepatoma cell HepG2 was more than that of normal hepatocytes L‐02, due to the presence of the cancer cell targeting unit Bio‐PEG (Figure 6d). Both in vivo and in vitro experiments demonstrated the remarkable targeting of Bio‐PEG‐CN/DNA nanocomposites to the hepatocellular carcinoma (HCC) sites. Consequently, Bio‐PEG‐CN/DNA nanocomposites could provide precise guidance in HCC gene therapy.

### 3.8 Gene therapy for tumors

**FIGURE 7:**
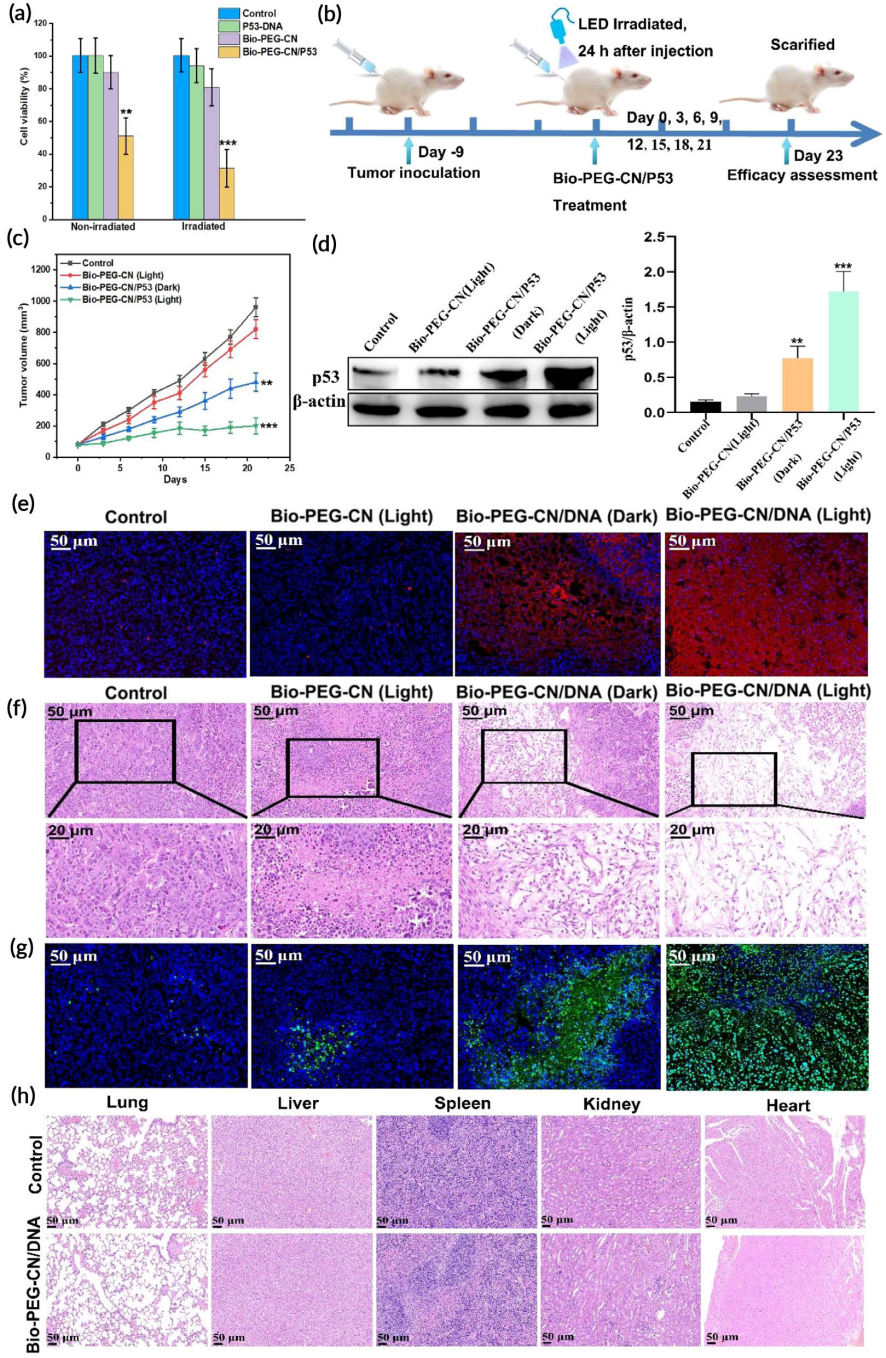
The viability of HepG2 cells incubated with and without LED irradiation, evaluated via the MTT assay. [Bio‐PEG‐CN] = 40 μg mL^−1^, [P53] = 10 μg mL^−1^. (b) Schematic diagram of mouse tumor treatment experiment. (c) Changes in tumor size after injection of PBS, Bio‐PEG‐CN nanosheets, and Bio‐PEG‐CN/*P53* with or without LED irradiation. (d) Western Blot analysis of P53 expression in tumor treated with *P53*‐loaded Bio‐PEG‐CN. (e–g) Histologic analysis of tumor sections of different treatment groups by (e) P53 immunofluorescence, (f) H&E staining, and (g) TUNEL assay. (h) H&E staining of lung, liver, spleen, kidney, and heart. **p < 0.01, ***p < 0.001 versus the control group (PBS). [Bio‐PEG‐CN] = 5 mg/kg, [*P53*] = 10 μg, wavelength: 401 nm, power density: 450 mW/cm^2^, irradiation time: 10 min.

To evaluate the therapeutic efficacy of this vector, the anti‐hepatoma effect of Bio‐PEG‐CN‐loaded *P53*‐DNA was first explored in HepG2 cells. As shown in Figure 7a, free *P53* without nanocarriers could not inhibit hepatoma cells growth due to its difficulty in uptake by tumor cells. Furthermore, when Bio‐PEG‐CN nanosheets were incubated with HepG2 cells, the ROS generated by these nanosheets had very limited inhibition of hepatoma cells growth after 10 min of LED irradiation, and the survival rate of HepG2 was still about 80%. In contrast, Bio‐PEG‐CN/*P53* complexes could inhibit HepG2 cells growth under dark conditions, indicating that Bio‐PEG‐CN/P53 could be taken up by tumor cells and exerted the antitumor effect. Especially under LED irradiation, HepG2 cells treated with Bio‐PEG‐CN/*P53* complexes exhibited better antitumor effect, and the cell viability decreased from 51% to 30%. The Bio‐PEG‐CN/*P53* light group had the most significant antitumor efficacy probably due to the following factors: First, Bio‐PEG‐CN nanosheets could promote the efficient entry of *P53* gene into tumor cells; Second, the ROS generated by Bio‐PEG‐CN under illumination could not only destroy the lysosomal structure to promote the escape of *P53* gene, but also exerted a certain effect of photodynamic therapy (PDT); Finally, the introduction of ester bonds could promote the reversible release of *P53* gene from nanosheets in the microenvironment of HCC, up‐regulated the expression of P53 protein, and synergized with PDT to exert anti‐HCC effects.^65^

In vivo antitumor evaluations were also performed to further explore the practical application of these functional Bio‐PEG‐CN/*P53* complexes (Figure 7b). After injection of Bio‐PEG‐CN nanosheets (5 mg/kg) every 2 days followed by LED irradiation, HCC volume increased from about 80 mm^3^ to 820 mm^3^, which was slightly smaller than that of the control group (Figure 7c). For the Bio‐PEG‐CN/*P53* dark group, HCC growth was observed to be inhibited, indicating that Bio‐PEG‐CN nanosheets successfully transfected the *P53* gene into the tumor site of nude mice, and realized effective reversible release of *P53*. Remarkably, HCC growth of the Bio‐PEG‐CN/*P53*‐administered group was basically inhibited after LED irradiation with a tumor inhibition rate of 80% (Figure 7c and Figure S13). It could be concluded that LED irradiation significantly induced the escape of more functional *P53* from lysosomes, then up‐regulated the tumor suppressor protein P53, and finally led to tumor apoptosis. To further determine the gene regulation effect of Bio‐PEG‐CN/*P53* in gene therapy, Western Blotting (WB) was used to detect the expression level of p53 protein in HCC tissue in vivo. The expression level of P53 protein in HCC tissues was significantly increased by Bio‐PEG‐CN/*P53* under LED irradiation, demonstrating the successful escape from lysosomes and efficient gene transfection of this vector under light irradiation (Figure 7d). In contrast, p53 protein was less expressed in other groups. Immunofluorescence assays were also used to evaluate the expression of P53 protein in various group. As shown in Figure 7e, the Bio‐PEG‐CN/*P53* light group exhibited more P53 positive‐staining cells compared with the control group, which was consistent with the WB results. The above results indicated that LED irradiation promoted the delivery of the *P53* by Bio‐PEG‐CN nanosheets. Meanwhile, a wide range of inflammatory cell infiltration and cell damage were observed in the Bio‐PEG‐CN/*P53* light group via H&E staining compared with other groups, further indicating that this nanocomposite exhibited the strongest antitumor effect under light (Figure 7e and Figure S14). The TUNEL (terminal deoxynucleotidyl transferase‐mediated deoxyuridine triphosphate nick end labeling) assay of tumor sections further revealed the degree of apoptosis in control and treatment groups (Figure 7g). It could be seen that the Bio‐PEG‐CN/*P53* light group maximally activated tumor apoptosis. In addition, body weight of nude mice did not change significantly and HE staining results also showed no obvious organ damage after Bio‐PEG‐CN/*P53* treatment due to the tumor targeting of the nanocomplexes (Figure 7h and Figure S15). Simultaneously, after mice were injected with Bio‐PEG‐CN/*P53* nanocomposites, no significant changes in biochemical parameters were found via blood biochemical analysis (Figure S16). Eventually, the concentration of phosphatase and aminotransferase in mice treated with Bio‐PEG‐CN/*P53* nanocomposites were not significantly changed (Figure S17), demonstrating that liver function of mice was also not damaged. In conclusion, the above in vivo pharmacodynamic experiments verified that the Bio‐PEG‐CN/*P53* nanocomposites realized effective gene therapy for HCC under LED illumination without obvious toxicity.

## 4 CONCLUSIONS

In summary, CN‐based non‐cationic hybrid nanocomposites Bio‐PEG‐CN/DNA were prepared for targeted gene therapy of HCC. Bio‐PEG‐CN nanosheets were demonstrated to generate ROS in tumor cells under LED irradiation, promoting the escape of Bio‐PEG‐CN/DNA nanocomposites from lysosomes. Moreover, Bio‐PEG‐CN/DNA nanocomposites were able to target HCC sites, reversibly released DNA in the tumor microenvironment, and completed DNA transfection and functional expression. After loading the tumor suppressor gene *P53*, Bio‐PEG‐CN/*p53* could achieve effective treatment of HCC by up‐regulating the expression of P53 protein under LED irradiation. Based on the remarkable in vitro and in vivo results, the Bio‐PEG‐CN nanosheets in this report represented just an example, confirming that the non‐cationic 2D nanosheets could achieve gene delivery and therapy through the interaction force between sheets instead of electrostatic interactions. This work provided a new idea for the development of non‐viral gene vectors.

## AUTHOR CONTRIBUTIONS

**Ming‐xuan Liu:** Conceptualization (lead); data curation (lead); formal analysis (lead); funding acquisition (lead); investigation (equal); methodology (lead); project administration (lead); resources (lead); software (lead); supervision (equal); validation (equal); visualization (lead); writing – original draft (lead); writing – review and editing (lead). **Li Xu:** Conceptualization (equal); data curation (equal); formal analysis (equal); methodology (equal); software (equal); validation (equal); writing – original draft (supporting); writing – review and editing (supporting). **Jia‐Yi Jiang:** Investigation (equal); methodology (supporting). **Hai‐Chen Dong:** Methodology (equal); validation (supporting). **Peng‐Fei Zhu:** Investigation (supporting); methodology (supporting). **Lei Cao:** Validation (supporting). **Jing Chen:** Funding acquisition (equal); project administration (supporting); resources (equal). **Xiao‐Ling Zhang:** Conceptualization (equal); funding acquisition (supporting); investigation (supporting); methodology (equal); project administration (equal); resources (equal); validation (supporting); writing – review and editing (supporting).

## CONFLICT OF INTEREST STATEMENT

The authors declare no conflicts of interest.

## Supporting information


**Data S1:** Supporting Information.

## Data Availability

The data that support the findings of this study are available in the supplementary material of this article.
