# Supplementary material for: Light controlled self‐escape capability of non‐cationic carbon nitride‐based nanosheets in lysosomes for hepatocellular carcinoma targeting stimulus‐responsive gene delivery
Source: Bioeng Transl Med. 2023 Jun 6;8(5):e10558. doi: 10.1002/btm2.10558 (PMC10486340; doi:10.1002/btm2.10558)
Supplement: Supplementary file 1 — Data S1: Supporting Information. [file BTM2-8-e10558-s001.docx]

Supporting Information

**Light controlled self-escape capability of non-cationic CN-based nanosheets in lysosomes for liver cancer targeting stimulus-responsive gene delivery**

Ming-Xuan Liu, *^a^ Li Xu,^b, c^ Jia-Yi Jiang,^a^ Hai-Chen Dong,^a^ Peng-Fei Zhu,^a^ Lei Cao, ^a^ Jing Chen, *^b, c^ Xiao-Ling Zhang*^a^

*^a^ School of Pharmacy, Nantong University, Nantong, 226001, China*

*^b^Institute of Translational Medicine, Medical College, Yangzhou University, Yangzhou 225001, Jiangsu, P. R. China.*

*^c^ Jiangsu Key laboratory of integrated traditional Chinese and Western Medicine for prevention and treatment of Senile Diseases, Yangzhou University, Yangzhou 225001, Jiangsu, P. R. China.*

*Correspondence: E-mail addresses: [mingxuanliu@ntu.edu.cn](mailto:mingxuanliu@ntu.edu.cn) (Ming-Xuan Liu), Zhangxiaoling@ntu.edu.cn (Xiao-Ling Zhang), and chenjing2018@yzu.edu.cn (Jing Chen).

Content

[1. Material characterization 3](#_Toc108452169)

[2. *In vitro* experiments 6](#_Toc108452170)

[3. *In vivo* experiments 10](#_Toc108452171)

1. Material characterization


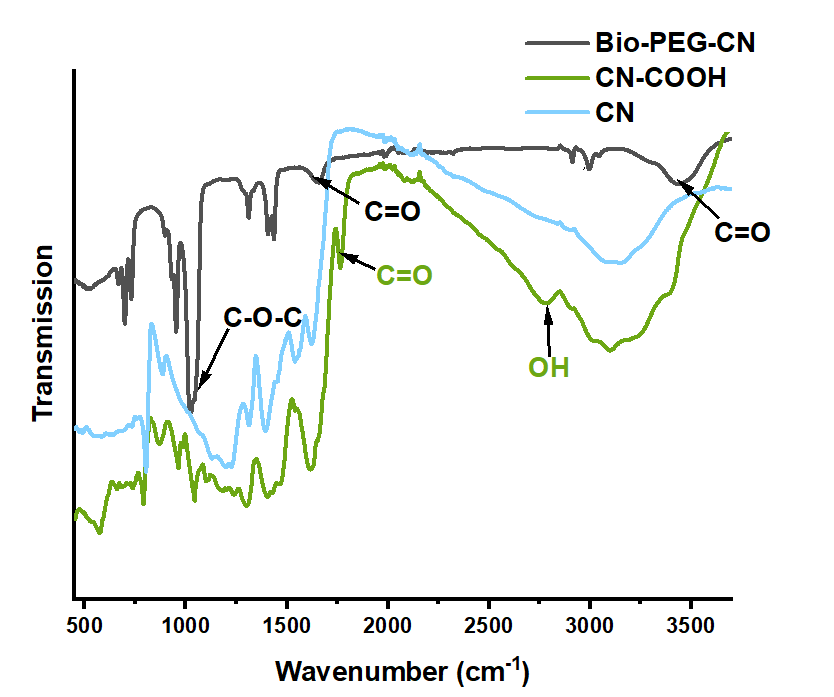


Figure S1. FT-IR spectra of CN, CN-COOH and Bio-PEG-CN nanosheets.


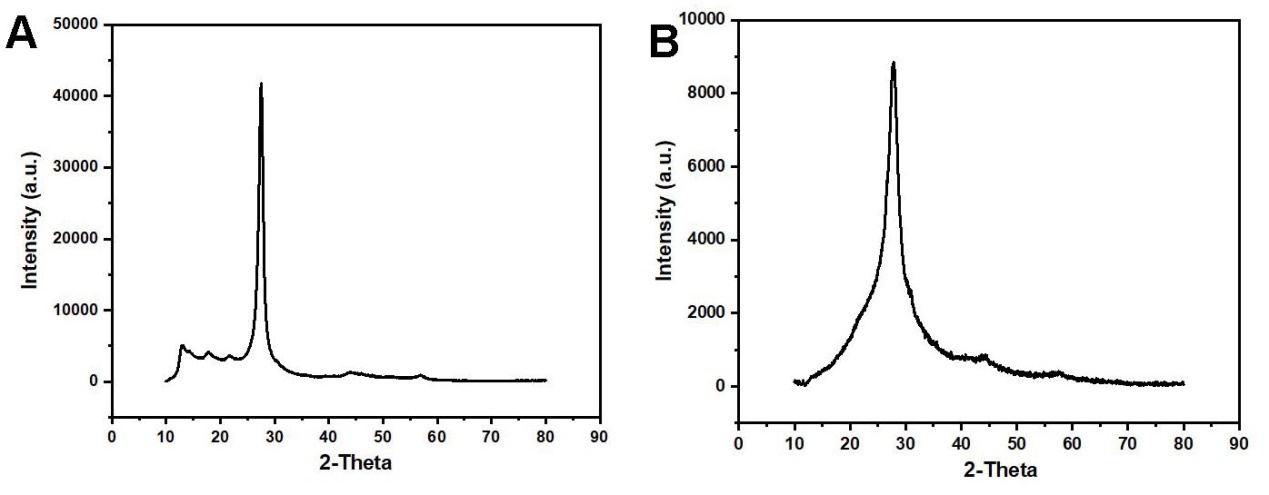


Figure S2. XRD patterns of (A) bulk CN and (B) Bio-PEG-CN nanosheets.


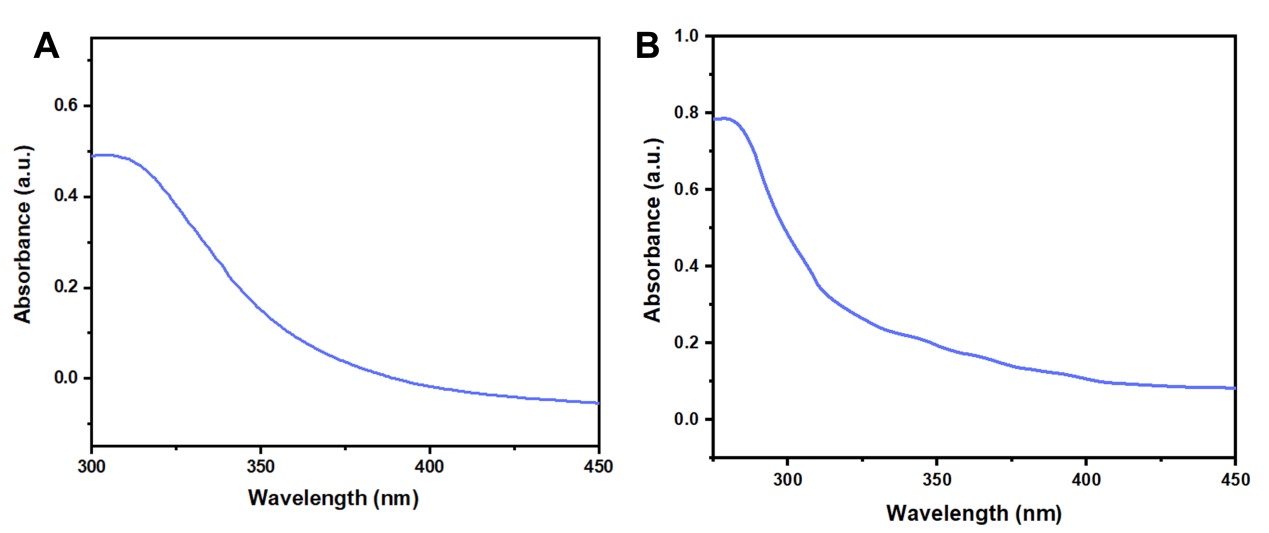


Figure S3. UV-Vis absorption of (A) CN-COOH and (B) Bio-PEG-CN nanosheets in water.


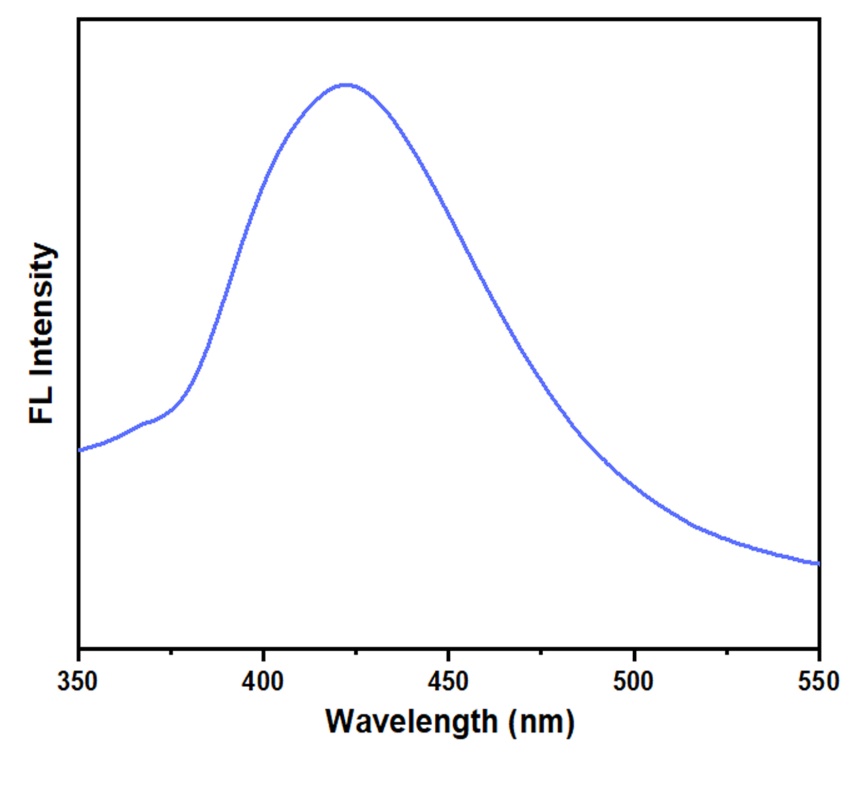


Figure S4. PL emission spectra of Bio-PEG-CN nanosheets in water.


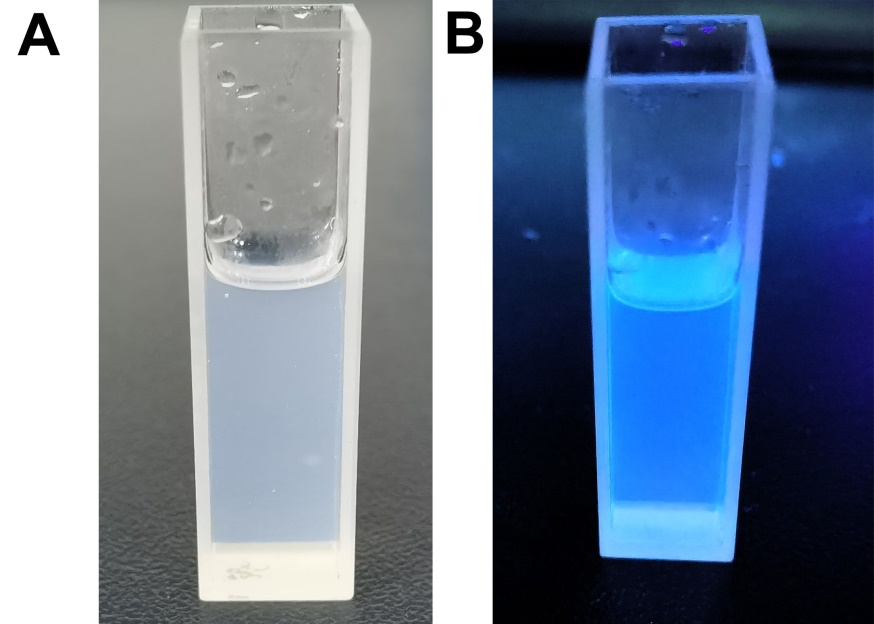


Figure S5. Photographs of Bio-PEG-CN nanosheets under (A) natural light and (B) hand-held UV lamp.


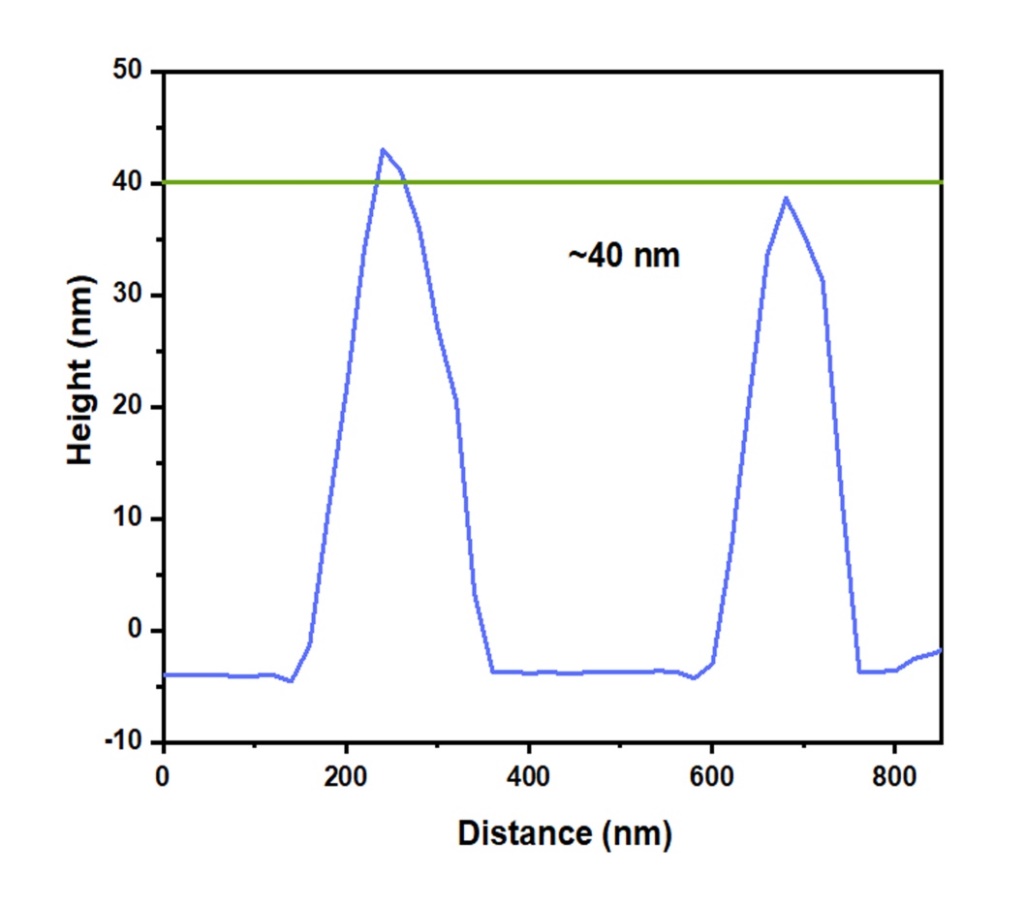


Figure S6. Thickness of Bio-PEG-CN nanosheets measured by AFM.

2. *In vitro* experiments


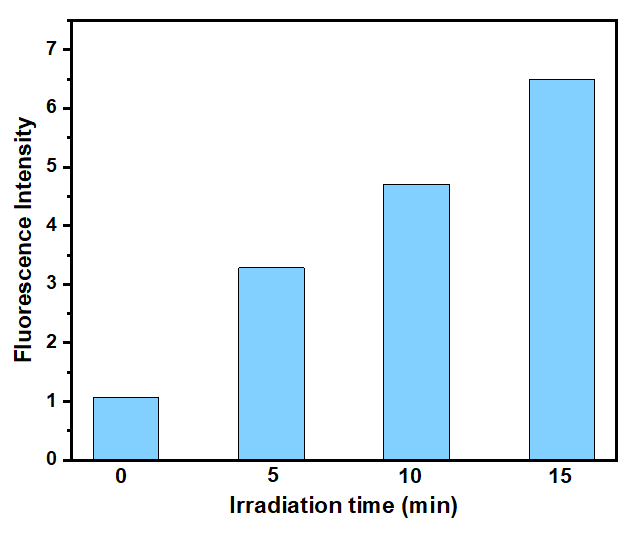


Figure S7. Analysis of DCFH fluorescence intensity in HepG2 after treatment with Bio-PEG-CN nanosheets. Power density: 450 mW/cm^2^, LED wavelength: 401 nm, [Bio-PEG-CN] = 40 µg/mL.


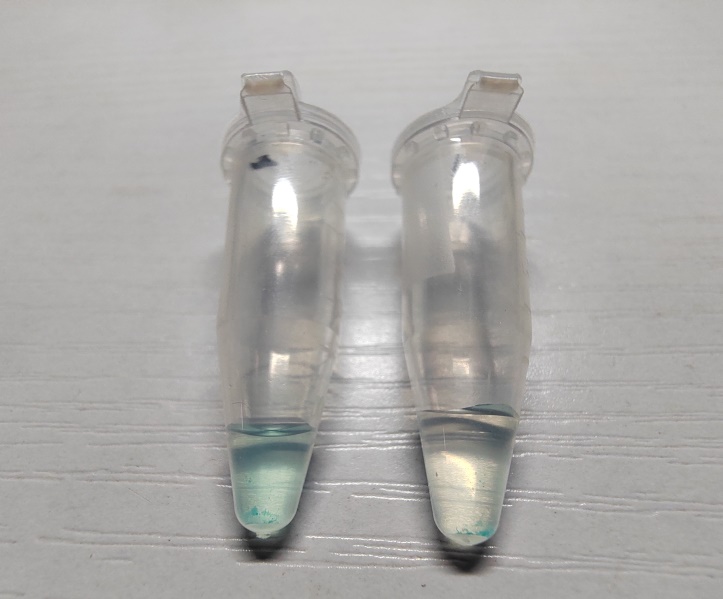


Figure S8. Optical images of DNA-Cy5 (left) and Bio-PEG-CN/DNA complex (right).


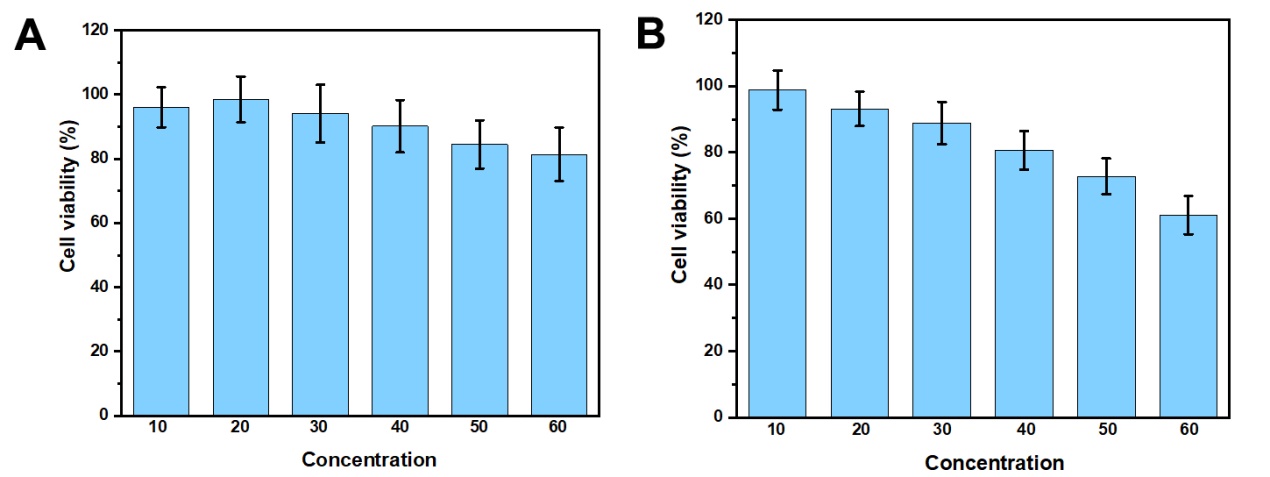


Figure S9. MTT experiments of Bio-PEG-CN nanosheets in (A) HepG2 and (B) L-02 cells.


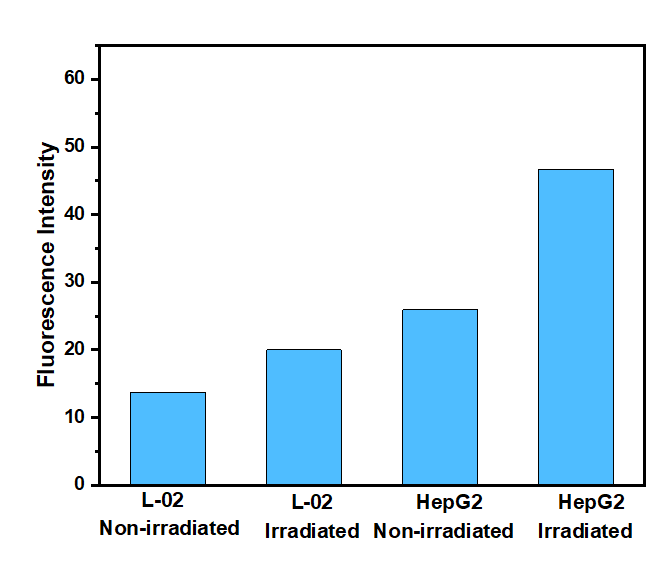


Figure S10. Fluorescence analysis of GFP expression in HepG2 cells. [Bio-PEG-CN] = 40 µg mL^-1^, [EGFP-DNA] = 10 μg mL^-1^, LED wavelength: 401 nm, power density: 450 mW/cm^2^ , irradiation time: 10 min.


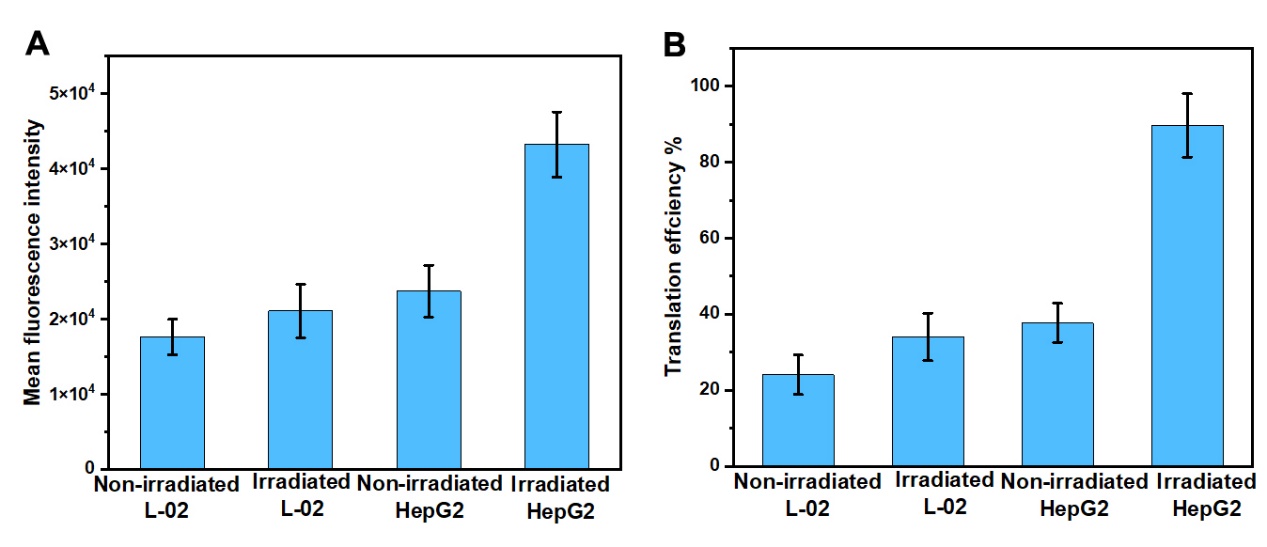


Figure S11. Evaluation of GFP expression in L-02 and HepG2 cells by cell flow cytometry: (A) GFP fluorescence intensity, (B) GFP transfection efficiency. [Bio-PEG-CN] = 40 µg mL^-1^, [EGFP-DNA] = 10 μg mL^-1^, LED wavelength: 401 nm, power density: 450 mW/cm^2^, irradiation time: 10 min.


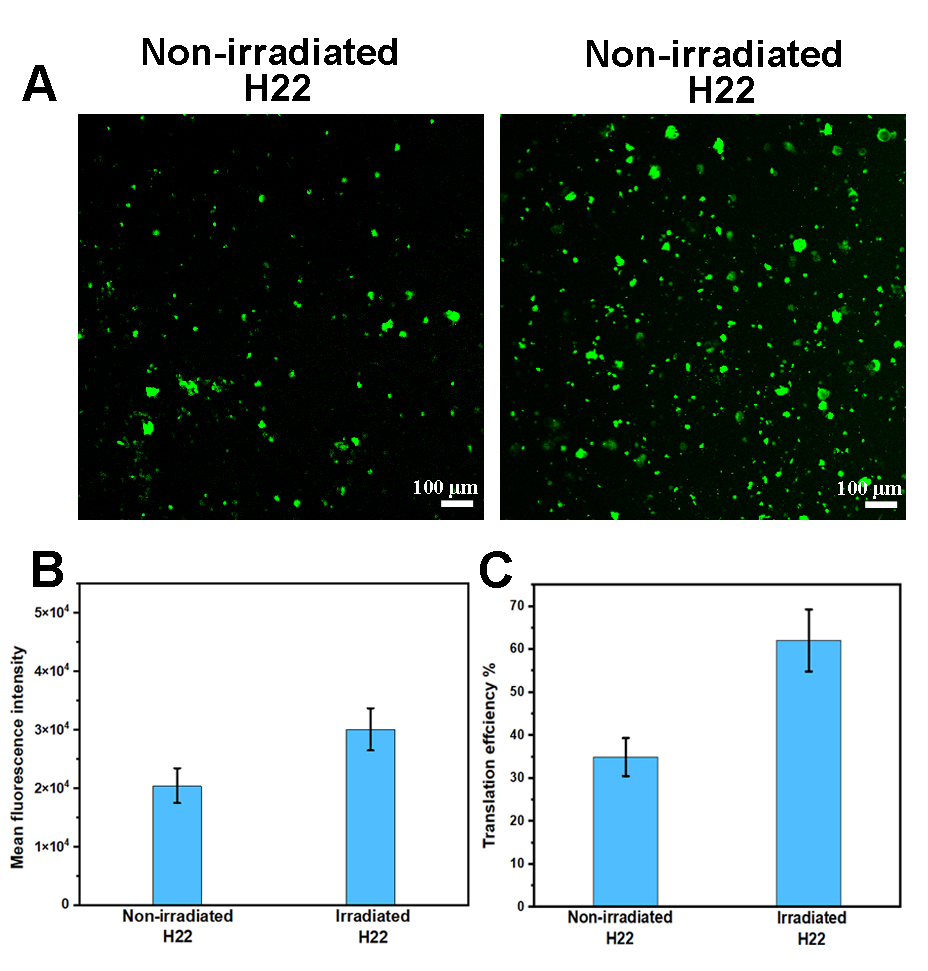


Figure S12. (A) GFP expression CLSM imaging (10×) of Bio-PEG-CN/DNA in H22 cells. CLSM imaging was captured at an excitation wavelength of 495–566 nm, scale bar: 100 μm. Evaluation of GFP expression in H22 cells by cell flow cytometry: (B) GFP fluorescence intensity, (C) GFP transfection efficiency. [Bio-PEG-CN] = 40 μg mL^-1^, [EGFP plasmid DNA] = 10 μg mL^-1^, irradiation time: 10 min, incubation time: 24 h.

3. *In vivo* experiments


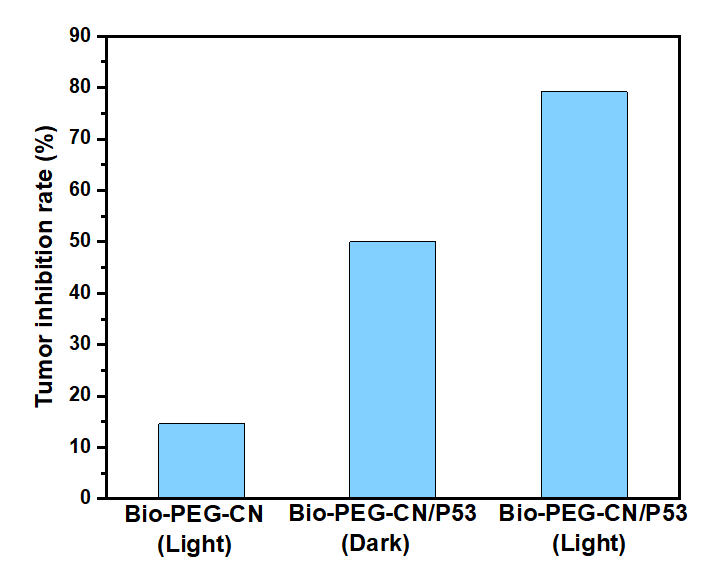


Figure S13. *In vivo* tumor inhibition rate study. [Bio-PEG-CN] = 5 mg/kg; [P53] = 10 μg; Wavelength: 401 nm; Power density: 450 mW/cm^2^, irradiation time: 10 min. Tumor inhibition rate (TIR) was calculated as the following equation: TIR (%) = 100×(average tumor weight of control group-average tumor weight of experimental group)/average tumor weight of control group.


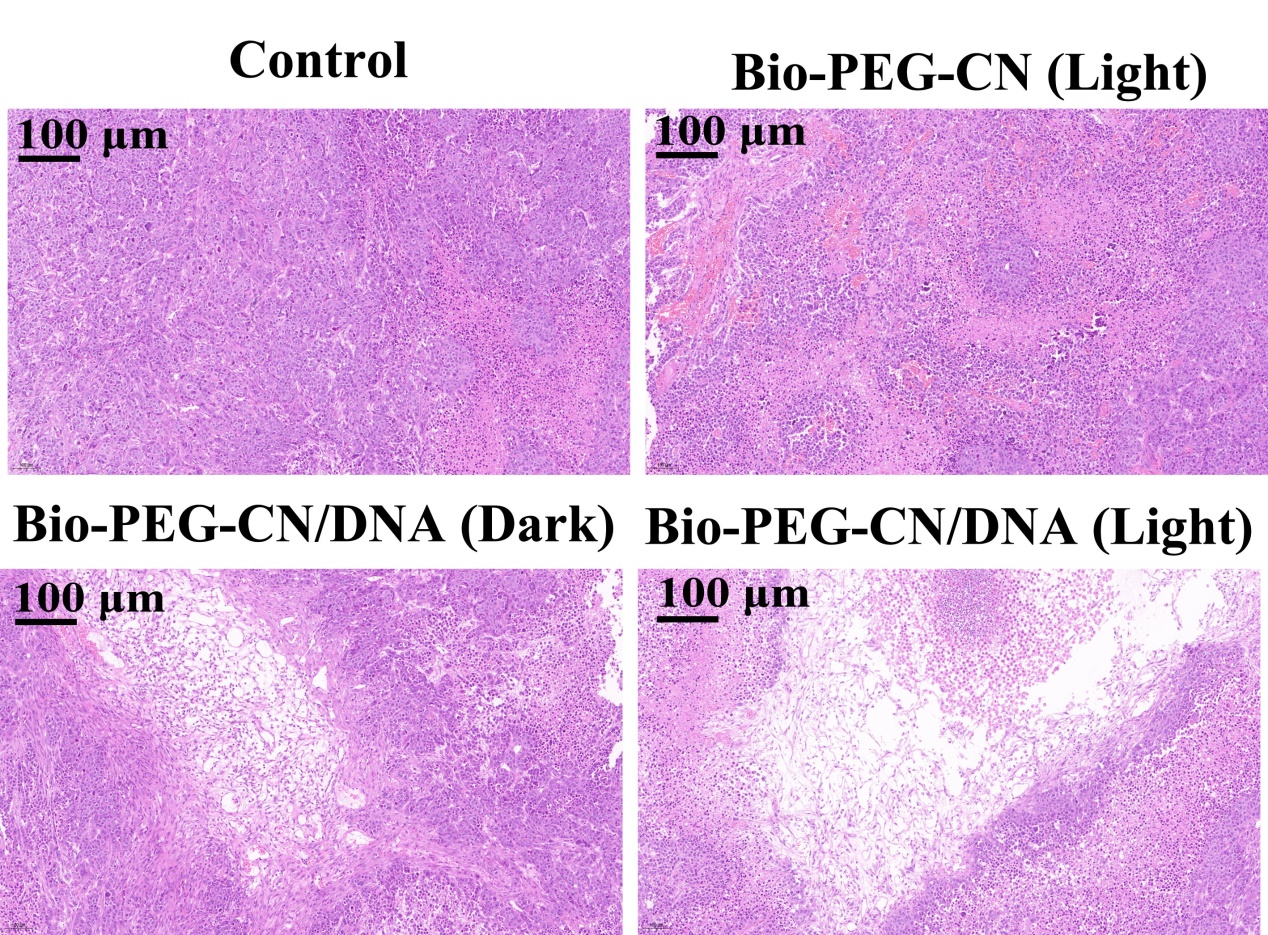


Figure S14. H&E staining of tumor tissues at 100 х, scale bar: 100 µm.


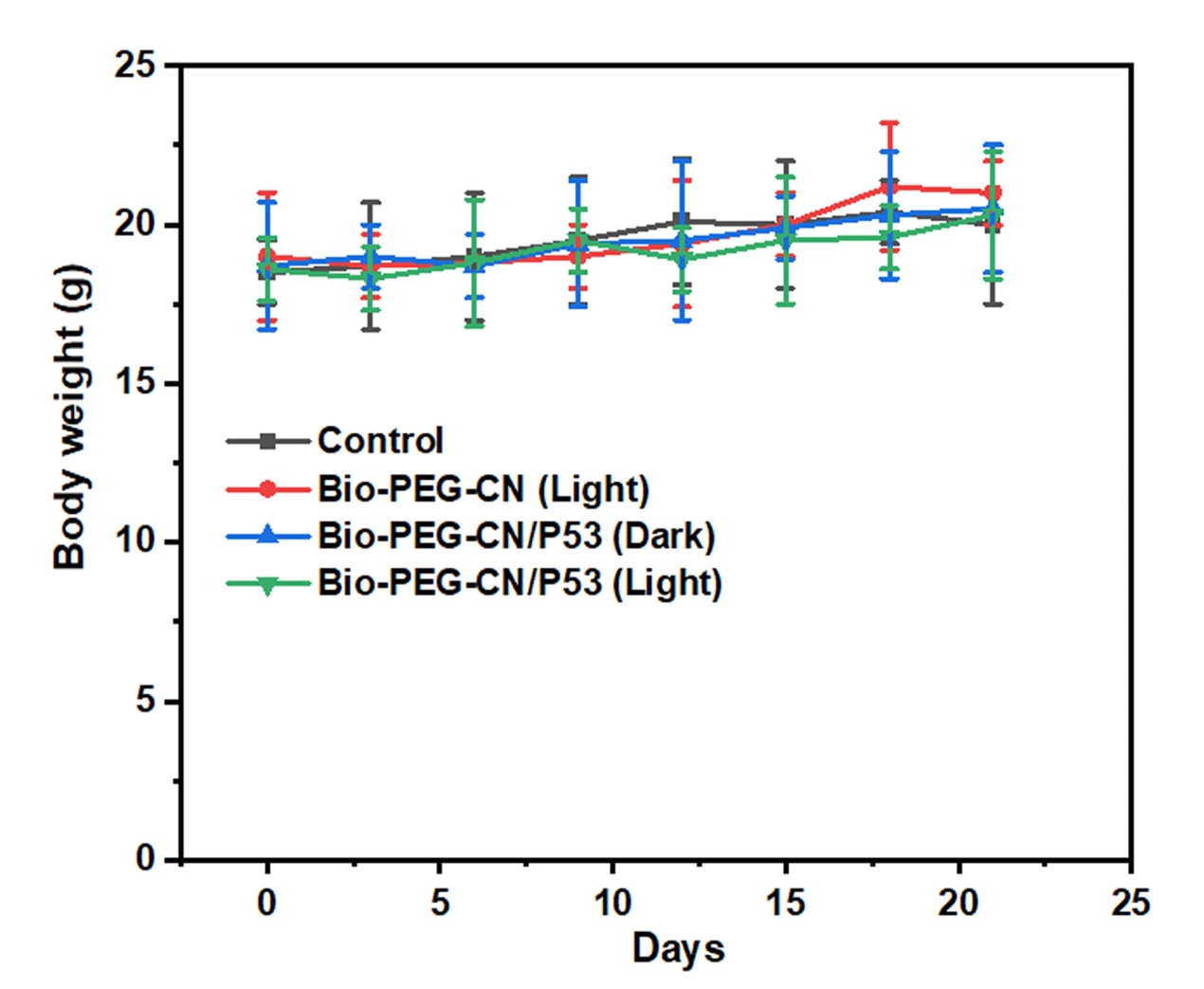


Figure S15. Body weight change of nude mice. [Bio-PEG-CN] = 5 mg/kg; [P53] = 10 μg; Wavelength: 401 nm; Power density: 450 mW/cm^2^, irradiation time: 10 min.


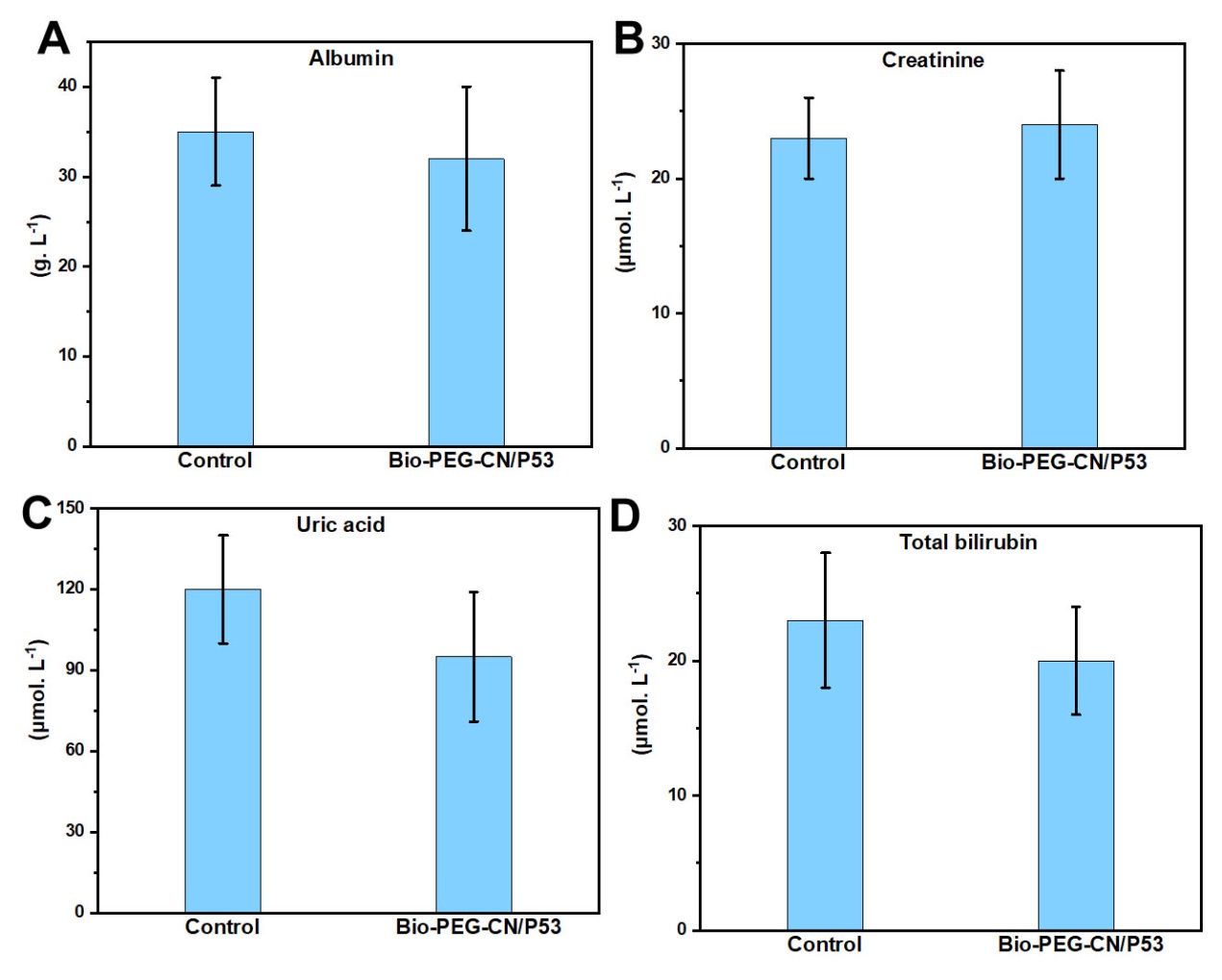


Figure S16. Analysis of blood chemistry in mice after Bio-PEG-CN/P53 treatment. Healthy mice without any treatment were used as controls. [Bio-PEG-CN] = 5 mg/kg; [P53] = 10 μg.


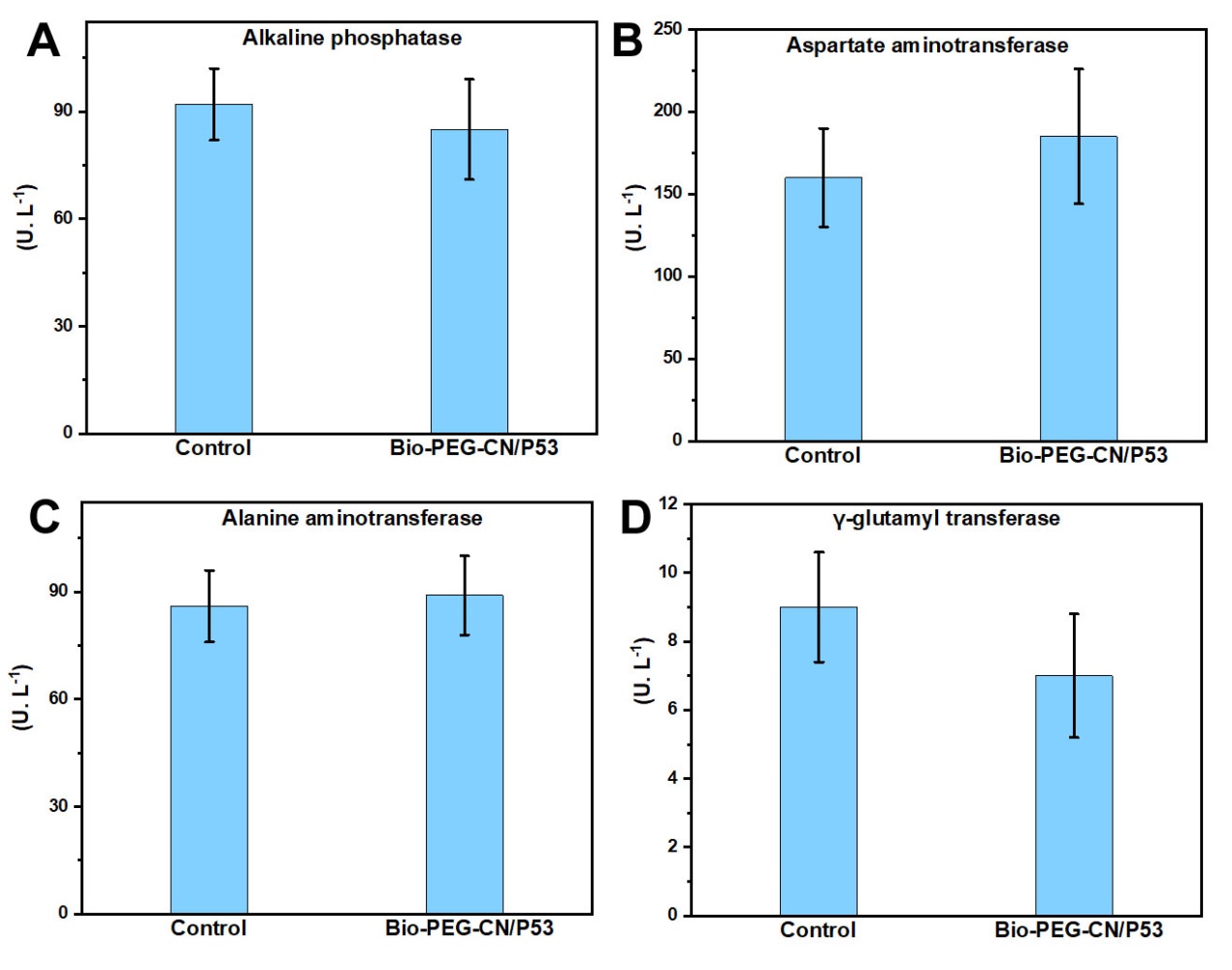


Figure S17. Analysis of liver function in mice after Bio-PEG-CN/P53 treatment. Healthy mice without any treatment were used as controls. [Bio-PEG-CN] = 5 mg/kg; [P53] = 10 μg.
